# Faster Sampling in Molecular Dynamics Simulations with TIP3P-F Water

**DOI:** 10.1021/acs.jctc.4c00990

**Published:** 2024-12-12

**Authors:** José Guadalupe Rosas Jiménez, Balázs Fábián, Gerhard Hummer

**Affiliations:** † Department of Theoretical Biophysics, Max Planck Institute of Biophysics, Max-von-Laue-Straße 3, 60438 Frankfurt am Main, Germany; ‡ IMPRS on Cellular Biophysics, Max Planck Institute of Biophysics, Max-von-Laue-Straße 3, 60438 Frankfurt am Main, Germany; § Institute of Biophysics, Goethe University Frankfurt, 60438 Frankfurt am Main, Germany

## Abstract

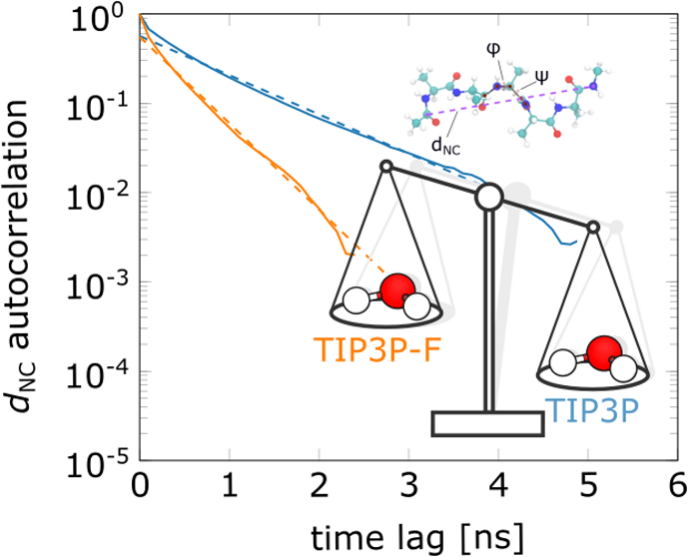

The need for short time steps currently limits routine atomistic molecular dynamics (MD) simulations to the microsecond time scale. For long time steps, the numerical integration of the equations of motion becomes unstable, resulting in catastrophic crashes. Here, we combine mass repartitioning and rescaling to construct a water model that increases the sampling efficiency in biomolecular simulations without compromising integration stability and with preserved structural and thermodynamic properties. The resulting “fast water” is then used with a time step as before in combination with standard force fields. The reduced water viscosity and faster diffusion result in proportionally faster sampling of the larger-scale motions in the conformation space of both solute and solvent. We illustrate this approach by developing TIP3P-F based on the popular TIP3P model of water. A roughly 2-fold boost in the sampling efficiency at minimal cost in accuracy is substantial and helps lower the energy impact of large-scale MD simulations. The approach is general and can readily be applied to other water models and different types of solvents.

## 1 Introduction

Despite enormous progress in algorithms and computing power, routine atomistic molecular dynamics (MD) simulations remain limited to the microsecond time scale. The reason is that the allowed time step for the time integration of the trajectories is set by the fastest molecular motions. For large time steps, time integration becomes unstable, primarily because deep particle collisions or fast bond vibrations result in large forces that at the next time step amplify the stability problem. The default time step of ∼2 fs in classical MD of biomolecules is below the stability limit so that deep collisions and the resulting catastrophic crashes are virtually impossible to occur in simulations of reasonable length for accessible system sizes.

A number of workarounds have been developed to stabilize time integration. For instance, bonds involving hydrogen atoms are often treated as rigid, which eliminates the highest-frequency motions.^1,2^ Also, the repartitioning of masses from heavy atoms to bonded hydrogen atoms has long been found to stabilize the time integration and make the use of longer time steps possible.^3^ Such schemes, known as hydrogen mass repartitioning (HMR), were systematically applied to hydrogen atoms in proteins^4^ and lipids,^5^ allowing stable simulations using 4 fs time steps. The statistical mechanical basis of all of these schemes is well established, as configuration space averages are independent of the masses in classical statistical mechanics.

Walser et al.^6^ rescaled the water masses to systematically modify the solvent viscosity. Lin and Tuckerman^7^ have treated the masses as free parameters to improve the efficiency of conformational sampling in protein simulations, establishing a hierarchy of adiabatic decoupling between solvent, side-chain, and backbone motions by decreasing the solvent and side-chain masses. This approach differs from mass repartitioning because, in practice, it tends to decrease the hydrogen masses along with the total solvent mass, whereas mass repartitioning increases hydrogen masses at constant total solvent mass. Nevertheless, both approaches aim to achieve larger particle displacements *per simulation step* without compromising the stability and accuracy of the numerical integration. In these and similar approaches, one should keep in mind that a uniform scaling of all masses in the system does not improve the sampling efficiency, because it simply amounts to a rescaling of time.

Lowering the solvent viscosity tends to speed up the rate of reactions in condensed phase, consistent with Kramers’ theory.^8^ In particular, there is ample experimental and computational evidence indicating that proteins exhibit faster internal motions and folding times as the solvent viscosity is reduced.^7,9−15^ Modifications of particle masses thus provide an opportunity both to stabilize the time integration of molecular motions and to improve the sampling efficiency of MD simulations.

Here, we develop a “fast water model” that fully retains all energetic and thermodynamic properties, yet substantially increases the sampling efficiency. We combine the two approaches of (1) quantifying the stability of MD time integration and (2) mass repartitioning with (3) the fact that in dilute (aqueous) solution, larger-scale molecular motions and thus sampling efficiency are largely determined by the viscosity of the solvent medium. To speed up the solute dynamics, we repartition and rescale the masses of water to reduce the viscosity without causing time integration instabilities. A simple analytical relation for the rate of catastrophic crashes *k*_crash_ in MD simulations as a function of the time step^16^ allows us to quantify the stability of the time integration. The resulting “fast water” is then used with a time step as before, to keep the time integration of the vibrational motions of, say, protein solutes stable. However, the reduced water viscosity and faster diffusion result in a proportionally faster sampling of the larger-scale motions in the conformation space of both solute and solvent. We illustrate this approach by developing the TIP3P-F model based on the modified version of the popular TIP3P model^17^ used with the CHARMM force field,^18^ but the scheme can readily be applied to different water models and other solvents.

The paper is organized as follows. First, we present the underlying theory for the effect of mass changes and the stability of MD time integration. After describing the simulation and analysis methods, we construct a water model optimized for sampling efficiency. To validate our model, we show that TIP3P-F preserves the key thermodynamic and structural properties of TIP3P water. We proceed to demonstrate that for small peptides and RNA segments, which can be sampled extensively in standard MD, relevant structural and energetic properties are preserved. We then show that TIP3P-F enhances the sampling by comparing autocorrelation functions of widely studied observables. Finally, we show applications of TIP3P-F to full-length proteins, and lipid bilayers, again comparing the results to runs with regular TIP3P. Overall, we find that using TIP3P-F with a conventional time step of 2 fs speeds up conformational sampling of biomolecules up to about a factor of 2 over regular TIP3P at no additional cost in computation.

## 2 Methods

### 2.1 Mass Scaling in MD Simulations

MD simulations are extensively employed to sample equilibrium configurational properties, i.e., quantities that depend on the particle positions, **r**, but not their momenta, **p**.^19^ When computing ensemble averages of these configurational properties, the classical partition function factorizes and, as a result, configuration space averages are independent of particle masses,
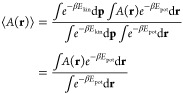
1where *A*(**r**) is the configurational property of interest, *E*_kin_ = **p**^T^*M*^–1^**p**/2 and *E*_pot_ are the total kinetic and potential energies, respectively, and β = 1/(*k*_B_*T*), *M* is the mass tensor and superscript *T* indicates the transpose. As a notable method that relies on eq 1, Metropolis Monte Carlo simulations of molecular systems accept or reject randomly proposed particle moves without referring to particle masses.^19^ Therefore, particle masses can be considered as *adjustable parameters* for the particular case of sampling equilibrium distributions of configurational properties. We note further that Newton’s equation of motion, i.e., mass times acceleration equals force,
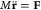
2is invariant if time and masses are scaled uniformly by factors *f* and *f*^2^, respectively. Expressed in terms of integration time steps and particle masses, we thus have the formal equivalence
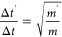
3

### 2.2 Mass Scaling of 3-Site Water

In MD simulations of biomolecules, the viscosity of the solvent is a major determinant of the speed not just of diffusion but also of conformational changes, and thus of the sampling efficiency. Scaling the masses of solvent molecules^6^ by a factor *f*^2^, i.e., *m*′ = *f*^2^m, changes the solvent viscosity by a factor *f*, η′ = *fη*.

Here, we consider TIP3P as a widely used 3-site water model.^17,18^ The masses of the oxygen and two hydrogen sites are *m*_O_ and *m*_H_, respectively, with a total mass of *m*_tot_ = *m*_O_ + 2*m*_H_. We modify the atomic masses in two consecutive steps. First, we repartition mass from the oxygen to the hydrogen atoms in a symmetric manner and, second, we scale the total mass *m*_tot_. The unmodified masses are *m*_O_ = 15.9994 and *m*_H_ = 1.008 with a total mass *M*_0_ = *m*_O_ + 2*m*_H_ = 18.0154 in atomic units (a.u.) of g/mol. Repartitioning by mass *m*_r_ decreases the oxygen mass to *m*_O,r_ = 15.9994 – *m*_r_ and increases the hydrogen masses to *m*_H,r_ = 1.008 + *m*_r_/2. Finally, after repartitioning, mass scaling changes the total mass from *M*_0_ to *m*_tot_, resulting in atomic masses of *m*_O_^′^ = (15.9994 – *m*_r_)(*m*_tot_/*M*_0_) and *m*_H_^′^ = (1.008 + *m*_r_/2)(*m*_tot_/*M*_0_), and a total mass of *m*_O_^′^ + 2 *m*_H_^′^ = *m*_tot_. We use total mass scaling to decrease the shear viscosity, and mass repartitioning to stabilize the time integration.

### 2.3 Stability of MD Simulations

In classical MD, the system of interest is propagated forward in discrete integration time steps, Δ*t*, according to Newton’s equations of motion, usually extended with a thermostat and barostat. The value of Δ*t* is a trade-off between computational efficiency (sampling of the configuration space) and accuracy (energy and momentum conservation). The time step commonly used in atomistic biomolecular simulations is Δ*t* ≈ 2 fs. Either increasing Δ*t* or decreasing the particle masses results in larger particle displacements *per single time step*, that is, in a single unit of computation. In MD simulations, the time step is usually chosen close to the limit of stable time integration to achieve near-optimal sampling.

We recently developed a kinetic model of the probability that a simulation with a given time step Δ*t* will crash during a given total simulation time.^16^ In this model, crashes are caused when a position update by the time integrator positions a fast-moving particle within the repulsive core of another particle. Large forces then result in numerical instabilities. The model describes crashes as Poisson-distributed events with exponential waiting times. For a system of point particles, we showed that the crash rate depends on the integration time step as
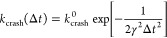
4where the prefactor *k*_crash_^0^ accounts for the specifics of the system and its size.^16^ The factor in the exponent is defined as 1/γ^2^ = *βm*Δ*x*_crit_^2^, with Δ*x*_crit_ the critical particle displacement in the model. The crash rate as a function of the particle mass *m* then becomes
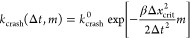
5Applied to water, if the integration time step and the mass repartitioning are kept constant, the crash rate is expected to decrease approximately exponentially with increasing total particle mass,
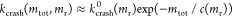
6Here, *c* is a constant that depends on *m*_r_. Complicating effects of rotation^16^ are ignored here.

We used a maximum-likelihood estimator of the crash rate^16^ in our MD simulations,
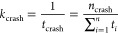
7where 0 ≤ *n*_crash_ ≤ *n* is the number of crashes observed in *n* independent simulations and *t*_*i*_ is either the time point of the crash in simulation *i* or its end point. Typically, *n* ranged from 40 at higher total masses to 400 at lower total masses. We assessed the uncertainty of the estimator using the standard error , which corresponds to the Cramér-Rao bound. For the exact definition of crashes, see ref (16).

### 2.4 Simulation Methods

We tested the TIP3P-F water model with reduced total mass and repartitioned oxygen mass in simulations of neat water, peptide, nucleic acid, protein, and lipid systems. For peptides, the N- and C-termini were blocked with methyl groups. The 5′ and 3′ termini of the polyribonucleotides were modeled as hydroxyl groups, without phosphate. All systems were prepared and solvated using the CHARMM-GUI server^20^ with the CHARMM36m force field parameters^21^ in combination with the CHARMM version of the TIP3P water model,^18^ which was kept rigid in all simulations using the SETTLE constraint algorithm.^1^ All simulations were performed using Gromacs 2020.1^22^ and the replicas and mass scaling were controlled through asyncmd.^23^ Two 10 ns-long equilibration runs were performed, first in the NVT ensemble at constant volume and temperature, and then in the NPT ensemble at constant pressure and temperature. The following production simulations in the NPT ensemble were run for times indicated in Supporting Table S1. All simulations were performed using a 2 fs time step at *T* = 310 K and *p* = 1 bar with the stochastic velocity rescaling thermostat^24^ (τ_t_ = 1 ps) and the Parrinello–Rahman barostat^25^ (τ_p_ = 5.0 ps). To estimate averages and autocorrelation times of selected collective variables, we simulated 10 replicas per system. For a more detailed list of simulation parameters, all input files can be accessed in zenodo.^26^

Simulations were analyzed using tools available in the GROMACS package as well as diffusion-GLS,^27^ custom MDAnalysis^28^ code, and updated unwrapping^29,30^ of water centers of mass, as implemented in qwrap (version 1.4; https://github.com/jhenin/qwrap). Curve fitting and statistical analyses were performed using SciPy.^31^ Notably, we computed the hydrogen-bonding properties and the deuterium order parameter^32^ using gmx hbond and gmx order, respectively. For details about autocorrelation analysis and for comparison of equilibrium distributions, see Supporting Text Sections 1.1 and 1.2.

## 3 Results

### 3.1 Construction of the TIP3P-F Model

#### 3.1.1 Crash Rate as a Function of Water Mass

**Figure 1:**
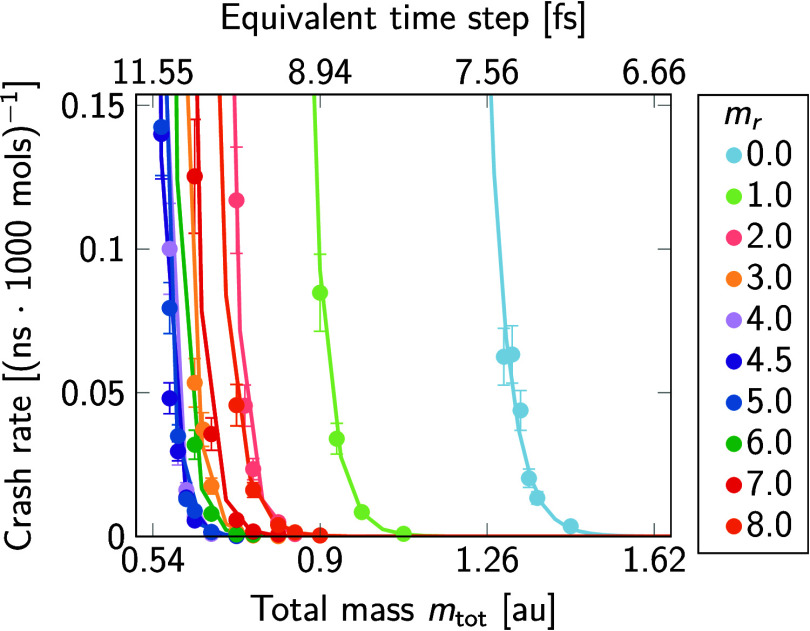
Crash rate *k*_crash_ in MD simulations of neat water as a function of the total mass *m*_tot_ of TIP3P water molecules with different repartitioned masses *m*_r_. The upper scale indicates the equivalent time step according to eq 3.

First, we systematically determined the stability of time integration for TIP3P water models with modified masses of the oxygen atom (*m*_O_) and hydrogen atoms (*m*_H_). We varied the repartitioned mass *m*_r_ from 0 to 8 g/mol in steps of 1 g/mol. For each repartitioning, we then varied the total mass in the regime of 0.5 < *m*_tot_ < 1.5 g/mol and collected the statistics of simulation crashes at a fixed time step Δ*t* = 2 fs. With the equivalence of time and mass scaling, eq 3, ignoring possible effects of thermostats and barostats, the mass rescalings correspond to probing time steps in the regime of 7 to 12 fs for TIP3P with unmodified total mass. The rate of crashing *k*_crash_ as a function of the total mass *m*_tot_ of the TIP3P water molecules with various values of *m*_r_ is shown in Figure 1. Results are shown for repartitioning *m*_r_ = 0 to 8 g/mol of the mass from the oxygen to the hydrogens. Repartitioning the mass from the oxygen atom to the two hydrogen atoms initially increases the integration stability. However, beyond *m*_r_ ≈ 4.5, the rate of crashing *k*_crash_ starts to increase with increasing *m*_r_ at a fixed value of *m*_tot_.

#### 3.1.2 Water Diffusion as a Function of Water Mass

We calculated the translational self-diffusion coefficient as a measure of the reciprocal fluid viscosity according to the Stokes–Einstein relation. To obtain continuous trajectories from the simulations under periodic boundary conditions, we used an unwrapping scheme^29^ that properly accounts for box-size fluctuations in constant-pressure MD. To determine diffusion coefficients from the unwrapped trajectories, we used a generalized least-squares-based estimator.^27^ We set the shortest lag time to 20 ps, resulting in excellent quality factors of the fits (*Q* ≈ 0.5). As we were only interested in relative changes due to *m*_r_ and the system size was not varied, the values of the diffusion coefficients were not corrected for effects of finite system size,^34,35^ except if stated otherwise.

**Figure 2:**
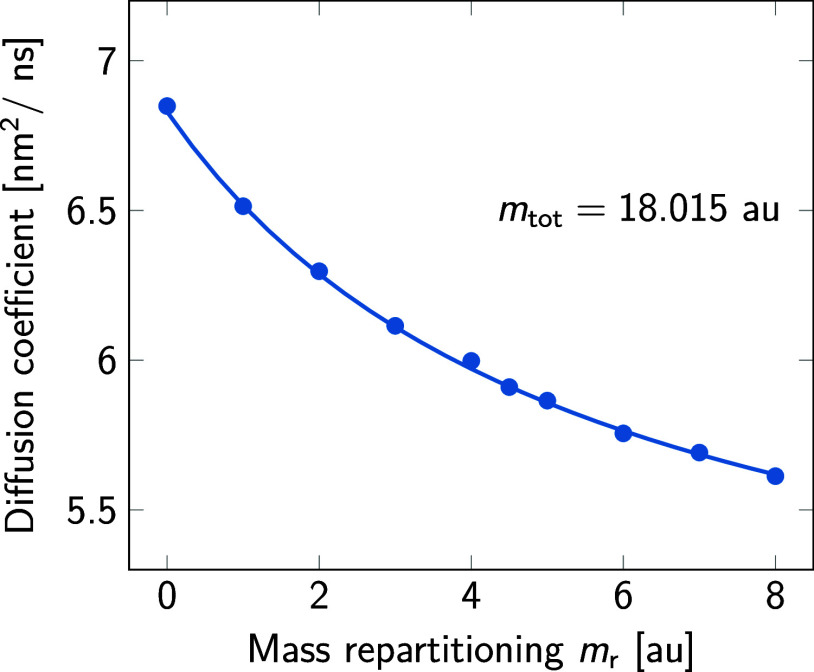
Self-diffusion coefficient of neat TIP3P water at *T* = 310 K and *p* = 1 bar as a function of the mass repartitioning, *m*_*r*_. Diffusion coefficients are not corrected for finite-size effects. The total mass *m*_tot_ of water molecules was unchanged. Data were averaged over two independent replicas, with differences between replicas being smaller than the symbol size. The line is a rational-function fit as a guide to the eye. For reference, finite-size corrected values of self-diffusion coefficients of TIP3P water at ambient pressure and 298 and 313 K have been reported as *D*_∞,298*K*_ = 6.22 nm^2^/ns and *D*_∞,313K_ = 7.48 nm^2^/ns,^33^ respectively, bracketing the value of 7.053 nm^2^/ns obtained here at an intermediate temperature of 310 K after finite-size correction^34^ (see Table 2).

Repartitioning mass *m*_r_ from O to H atoms at fixed total mass slows down water diffusion (Figure 2). The diffusion coefficient decreases by ≈14% from *m*_r_ = 0 to the most stable *m*_r_ = 4.5. This finding is consistent with earlier results by Feenstra et al.^3^

**Figure 3:**
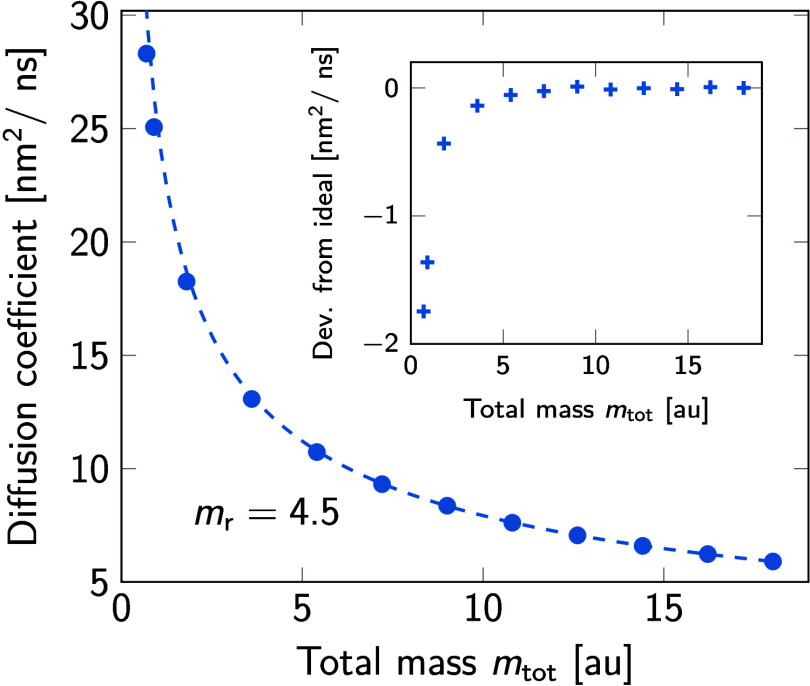
Self-diffusion coefficient of neat TIP3P water at *T* = 310 K and *p* = 1 bar as a function of the total mass *m*_tot_ of water molecules for fixed mass repartitioning *m*_r_ = 4.5 (symbols). The line corresponds to ideal scaling of the diffusion coefficient (line) according to eq 8. Results are not corrected for finite-size effects. Data point are averages over two independent replicas. The differences between the two runs are smaller than the symbols. The inset shows the deviation of the diffusion coefficient from the ideal scaling. For reference, finite-size corrected self-diffusion coefficients of TIP3P water have been reported as *D*_∞,298K_ = 6.22 nm^2^/ns and *D*_∞,313K_ = 7.48 nm^2^/ns,^33^ bracketing the value of 7.05 nm^2^/ns obtained here at an intermediate temperature of 310 K after finite-size correction.^34^

We also tested for possible deviations of the calculated diffusion coefficients from ideal mass scaling by fixing *m*_r_ and varying *m*_tot_. From the invariance of Newton’s equation of motion, eq 2, the self-diffusion coefficient of neat water should scale as
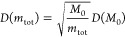
8with total mass for fixed *m*_r_. As shown in Figure 3, the expected mass scaling is quite accurately captured by the MD simulations of TIP3P water with modified total mass and *m*_r_ = 4.5. However, as the total mass approaches zero, the increasingly significant friction of the thermostat and possible errors in the time integration lead to less-than-ideal acceleration of the water self-diffusion, as shown in the inset.

**Figure 4:**
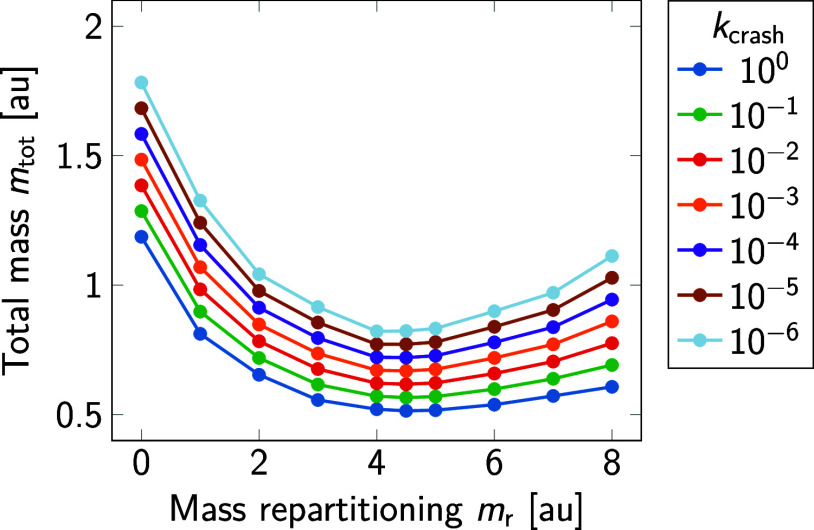
Rate of crashes of MD simulations of neat water as a function of total mass *m*_tot_ (*y* axis) and repartitioned mass *m*_r_ (*x* axis). Contour lines correspond to fixed crash rates *k*_crash_ in units of (ns × 1000 molecules)^−1^ as computed using eq 6 and the parameters reported in Table S2.

We note that in the development of “fast water” we are not interested in the most stable repartitioning *per se*, but rather in the fastest diffusing water at a fixed crash rate *k*_crash_. In Figure 4, we show lines of constant crash rate in the plane of *m*_r_ and *m*_tot_, as obtained by fitting eq 6 to the data in Figure 1 and then evaluating the fits at constant *k*_crash_. To zoom in on the region of high integration stability, we performed additional simulations at *k*_crash_ ≈ 3.1 × 10^–4^ (ns × 1000 molecules)^−1^. For masses (*m*_tot_, *m*_r_) = (0.766, 3.0), (0.696,4.0) and (0.694,4.5), we obtained diffusion coefficients of 27.52 ± 0.01, 28.44 ± 0.02, and 28.31 ± 0.04 nm^2^/ns, respectively. Therefore, for our purpose, the optimal value for mass repartitioning is *m*_r_ = 4.0, as it results in the fastest diffusing water for fixed *k*_crash_. Interestingly, the same amount of mass is repartitioned from carbon to methyl hydrogen atoms in force fields employing HMR.^4,5^ The masses of the TIP3P and TIP3P-F water models are presented in Table 1 for reference.

**Table 1:** Masses of the Original TIP3P^17,18^ and the Modified TIP3P-F Model (with *m*_tot_ = 1.116 and *m*_r_ = 4) Developed in This Work

water model	*m*_O_ [au]	*m*_H_ [au]	*m*_tot_ [au]
TIP3P	15.9994	1.008	18.0154
TIP3P-F	0.744	0.186	1.116

While developing the water model required us to use parameters that result in a measurable number of crashes, for production purposes, we used a more conservative value of *m*_tot_ = 1.116 g/mol; hence, *k*_crash_ was on the order of 10^–11^ (ns × 1000 molecules)^−1^. For a system of 1 million water molecules, we thus expect one crash in about 100 ms of MD.

**Figure 5:**
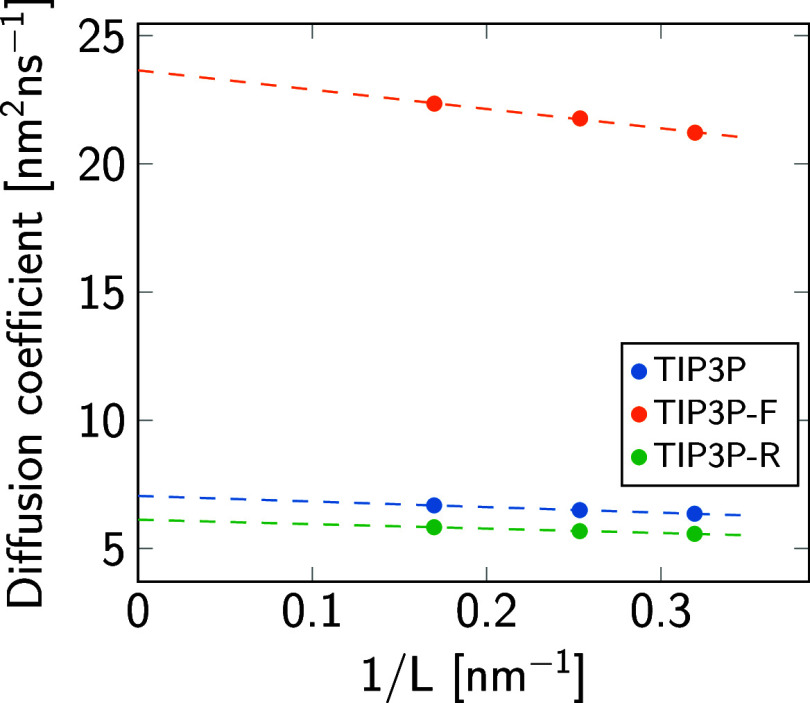
System size dependence of the self-diffusion coefficient for the original (TIP3P), repartitioned (TIP3P-R), and fast water model (TIP3P-F). The intercepts of straight-line fits^34^ at infinite box size (1/*L* → 0) correspond to the finite-size-corrected diffusion coefficient. The slope is proportional to the reciprocal of the shear viscosity.

The diffusion coefficient of the TIP3P-F model with repartitioned and scaled masses was corrected for finite-size effects following the method by Yeh and Hummer.^34^ Three cubic water boxes with increasing side length *L* were prepared in CHARMM-GUI^20^ and simulated in the NPT ensemble for 100 ns using an isotropic barostat. Uncorrected diffusion coefficients as a function of the inverse box length are presented in Figure 5. Least-squares fitting to a straight line and extrapolation to infinite size (1/*L* → 0) yielded the results in Table 2. As expected, mass transfer from the oxygen to the hydrogen atoms decreases the diffusion coefficient, while mass repartitioning combined with scaling increases the finite-size-corrected diffusion coefficient by a factor of 3.35 with respect to the original TIP3P model. Also, the slope in Figure 5 indicates a significant decrease in water viscosity^34^ by a factor of 0.293 consistent with a ratio of 1/0.298 of the extrapolated diffusion coefficients *D*_∞_ (Table 2).

**Table 2:** Effect of the Water Mass Repartitioning (*m*_r_ = 4.0 g/mol) and Mass Rescaling (*m*_tot_ = 1.116 g/mol) on the Diffusion Coefficient *D*_∞_ after Finite-Size Corrections for the Original (TIP3P), Repartitioned (TIP3P-R), and Fast Water Model (TIP3P-F)

water model	*D*_∞_ [nm^–2^ s^–1^]
TIP3P	7.053
TIP3P-R	6.125
TIP3P-F	23.648

### 3.2 Validation and Sampling Efficiency of the TIP3P-F Model

#### 3.2.1 Water Structure and Energetics

**Figure 6:**
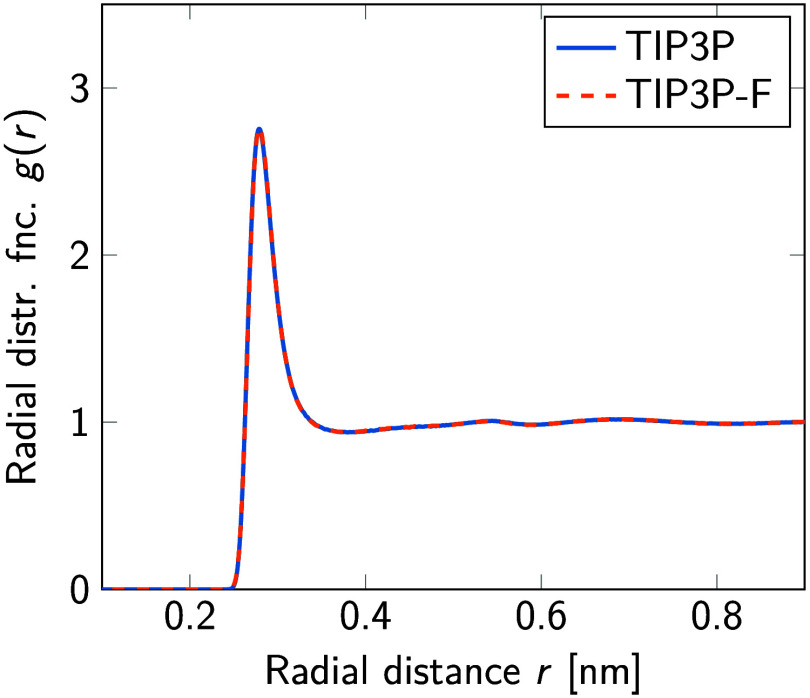
Oxygen–oxygen radial distribution function of TIP3P^17^ (solid blue line) and TIP3P-F water model (red dashed line) in neat water at *T* = 310 K and 1 bar pressure.

For neat water (Table 3), the combined effect of repartitioning the masses (*m*_r_) and decreasing the total mass (*m*_tot_) by a factor of ≈26 does not substantially change the average potential energy per molecule, ⟨*E*_pot_⟩/*N*, the average volume, ⟨*V*⟩, or the number of hydrogen bonds, ⟨#H-bonds⟩. However, decreasing the total mass substantially increases the self-diffusion coefficient, *D*, of the water molecules and decreases the lifetime,^36^ τ_H–bond_, of hydrogen bonds. Even though the change in the potential energy is minor, it is statistically significant. Similar systematic tendencies in the potential energy as a function of the time step have been reported previously.^4,37^ Additionally, as Figure 6 shows, our approach leaves the radial distribution functions unchanged.

**Table 3:** Comparison of Potential Energy, Volume, Number of Hydrogen Bonds, and Their Lifetime between the Original TIP3P^17^ and TIP3P-F Water Models in NPT Simulations of 31859 Molecules.^a^

water model	⟨*E*_pot_⟩/*N* [kJ/mol]	⟨*V*⟩ [nm^3^]	⟨#H-bonds⟩	τ_H–bond_ [ps]
TIP3P	–40.322 (0.001)	955.41 (0.02)	3.30 (0.01)	1.93 (0.07)
TIP3P-F	–40.400 (0.001)	953.15 (0.02)	3.31 (0.01)	1.19 (0.02)

a The reported uncertainties (except for τ_H–bond_) in parentheses correspond to SEM and were calculated with block average analysis. The error in τ_H–bond_ is the χ^2^ value of the fit. The average number of hydrogen bonds matches well the literature value of 3.36 at *T* = 298.15 K,^38^ while τ_H–bond_ closely follows the values reported for other water models at *T* = 298 K.^39^

#### 3.2.2 Kinetic Energy Repartitioning

Recently, Asthagiri and Beck^40^ showed that long time steps in molecular dynamics simulations of rigid water models lead to noticeable violations of the equipartition of kinetic energy. Since mass rescaling is equivalent to an increase in the integration time step, we expect similar effects for the TIP3P-F model presented here. We performed additional simulations using the protocol and temperature (298.15 K) reported by Asthagiri and Beck,^40^ and calculated the temperatures associated with the translational (*T*_trs_) and rotational (*T*_rot_) degrees of freedom, using the original TIP3P model, the model with hydrogen mass repartition-only (TIP3P-R; *m*_*r*_ = 4, *m*_tot_ = 18.0154), and the fast water model (TIP3P-F). Asthagiri and Beck^40^ tested equipartition with a velocity Verlet integrator combined with either the velocity rescaling or Langevin thermostat. Here, we used the velocity Verlet integrator with the velocity rescaling thermostat (VV-VR), and the Leap Frog integrator with velocity rescaling (LF-VR) or Langevin thermostats (LF-L). Figure S1 shows our results for all three integrator combinations and all three water models. While increasing the time step leads to an increase in *T*_trs_ and a decrease in *T*_rot_ in the case of VV-VR, the combinations LF-VR and LF-L showed the opposite tendency. Systems with only repartitioned hydrogen masses (without rescaling) are basically unaffected by the integration time step, yielding *T*_trs_ and *T*_rot_ close to the target value. By contrast, deviations between the two temperatures are amplified with the TIP3P-F model as the integration time step increases. In their work, Asthagiri and Beck^40^ attribute this behavior to the short relaxation times of the rotational velocity, comparable to the time scale of vibrational modes present in flexible water models. Therefore, such deviations in the energy repartition are inherent to the rigid body description of water and further work is required to elucidate its effect on the dynamics and thermodynamics of biological systems. In simulations using algorithms that rely on the sampling of canonical partition functions, such as replica exchange, small deviations from the Boltzmann distribution can be amplified.^41^

### 3.3 Validation and Sampling Efficiency for Peptides

**Figure 7:**
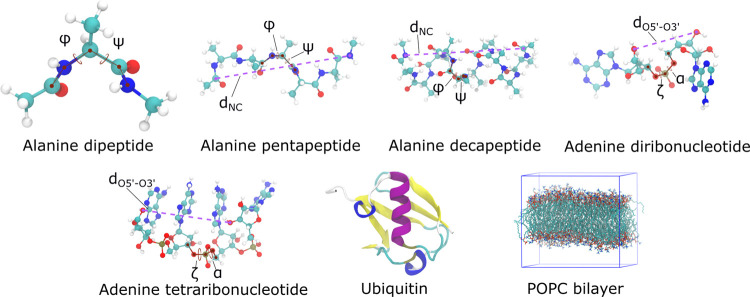
Illustration of the solutes tested in MD simulations with TIP3P-F water. Collective variables used to analyze the trajectories are indicated.

To demonstrate the increase in sampling efficiency and the preservation of equilibrium properties by the fast water model, we performed molecular dynamics simulations of alanine-based peptides. In particular, the alanine dipeptide serves as a minimal system to mimic the backbone dynamics of a protein. Additionally, the alanine penta- and decapeptides can exhibit transitions between secondary structure elements commonly found in folded proteins. We selected the Ramachandran dihedral angles of the central residues to describe the structure of the polypeptides, as illustrated in Figure 7. Additionally, we also used the distance between the N-terminal and C-terminal atoms (end-to-end distance) as a collective variable to characterize the penta- and decapeptides. Free energies were estimated from histogram counts,
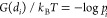
9where *d*_*i*_ is the value of the collective variable at the bin center and *p*_*i*_ is the proportion of observed samples in bin *i* for equal-sized bin widths.

**Figure 8:**
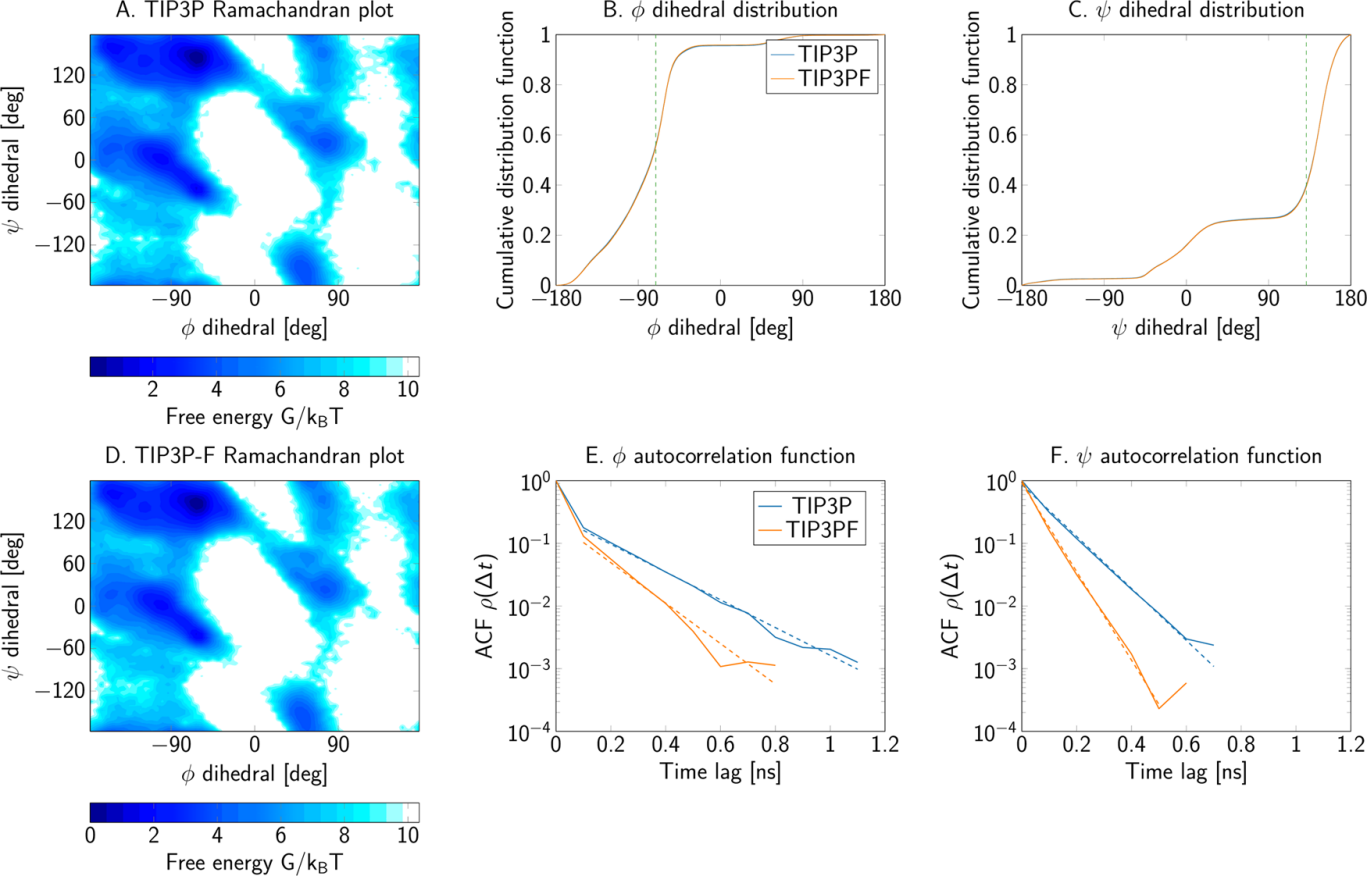
Dihedral angles of alanine dipeptide as sampled in simulations with the (A–C) TIP3P and (D–F) TIP3P-F water models. (A, D) Free energy as a function of the dihedral angles. (B, C) Cumulative distribution functions of dihedrals in TIP3P (blue) and TIP3P-F water (orange). The dashed vertical lines show where the two cumulative distributions are furthest apart. (E, F) Van der Spoel–Berendsen^42^ autocorrelation functions of the dihedrals for the two water models.

For alanine dipeptide, free energy plots as a function of the Ramachandran angles are depicted in Figure 8A,D comparing both water models. Alongside the Ramachandran maps, the marginal cumulative distribution functions of each dihedral (Figure 8B,C) demonstrate that changes in the water mass have a negligible effect on the final equilibrium distribution. Furthermore, the dihedral angle order parameters, *S*_D_^2^ (see the Supporting Text Section 1.1 for the definition), for both Ramachandran angles reported in Table 4, are in good agreement.

**Table 4:** Dihedral Order Parameters and Extensions for Alanine Dipeptide (AlaDip), Ala_5_, and Ala_10_^a^

peptide	collective variable	water model	average (SE)	τ̂_int_ (SE) [ns]	τ̂_exp_ (SE) [ns]
AlaDip	*S*_D_^2^(ϕ)	TIP3P	0.594 (0.001)	0.092 (0.001)	0.189 (0.006)
		TIP3P-F	0.601 (0.001)	0.072 (0.001)	0.118 (0.008)
	*S*_D_^2^(ψ)	TIP3P	0.293 (0.001)	0.100 (0.001)	0.104 (0.002)
		TIP3P-F	0.299 (0.001)	0.070 (0.001)	0.061 (0.001)
Ala_5_	*d*_NC_ [nm]	TIP3P	1.270 (0.001)	0.607 (0.010)	0.989 (0.015)
		TIP3P-F	1.275 (0.001)	0.281 (0.003)	0.448 (0.007)
	*S*_D_^2^(ϕ)	TIP3P	0.639 (0.002)	0.254 (0.003)	0.710 (0.006)
		TIP3P-F	0.643 (0.001)	0.141 (0.001)	0.351 (0.004)
	*S*_D_^2^(ψ)	TIP3P	0.315 (0.003)	0.582 (0.011)	0.923 (0.016)
		TIP3P-F	0.330 (0.002)	0.268 (0.003)	0.441 (0.010)
Ala_10_	*d*_NC_ [nm]	TIP3P	2.150 (0.005)	2.374 (0.085)	14.877 (0.082)
		TIP3P-F	2.173 (0.003)	1.291 (0.051)	9.260 (0.076)
	*S*_D_^2^(ϕ)	TIP3P	0.642 (0.002)	0.833 (0.027)	20.640 (0.546)
		TIP3P-F	0.648 (0.001)	0.401 (0.009)	8.342 (0.264)
	*S*_D_^2^(ψ)	TIP3P	0.149 (0.006)	5.173 (0.288)	16.734 (0.044)
		TIP3P-F	0.164 (0.004)	2.468 (0.093)	8.289 (0.045)

a Averages and relaxation times τ̂_int_ and τ̂_exp_ are listed with SE in parentheses.

In addition to the free energy maps of Figure 8, we also present their difference, *G*_TIP3P_ – *G*_TIP3P-F_, in Figure S2. It is clear from these differences that the fast water model preserves the positions of the energy minima in the Ramachandran map. Also, the relative energies between minima are practically unchanged, indicating that the populations of the alanine dipeptide conformers observed in the simulations with both water models are comparable. Notably, Figure S2 also shows that the largest deviations from the original TIP3P model appear at the edges of the distribution, as demonstrated by superposition of the isocontours of the Ramachandran map using the reference TIP3P model. Conformations in these regions of the energy surface are relatively poorly sampled due to their low equilibrium probability. Indeed, the sign of the difference shows no discernible pattern in the Ramachandran representation (Figure S2).

**Figure 9:**
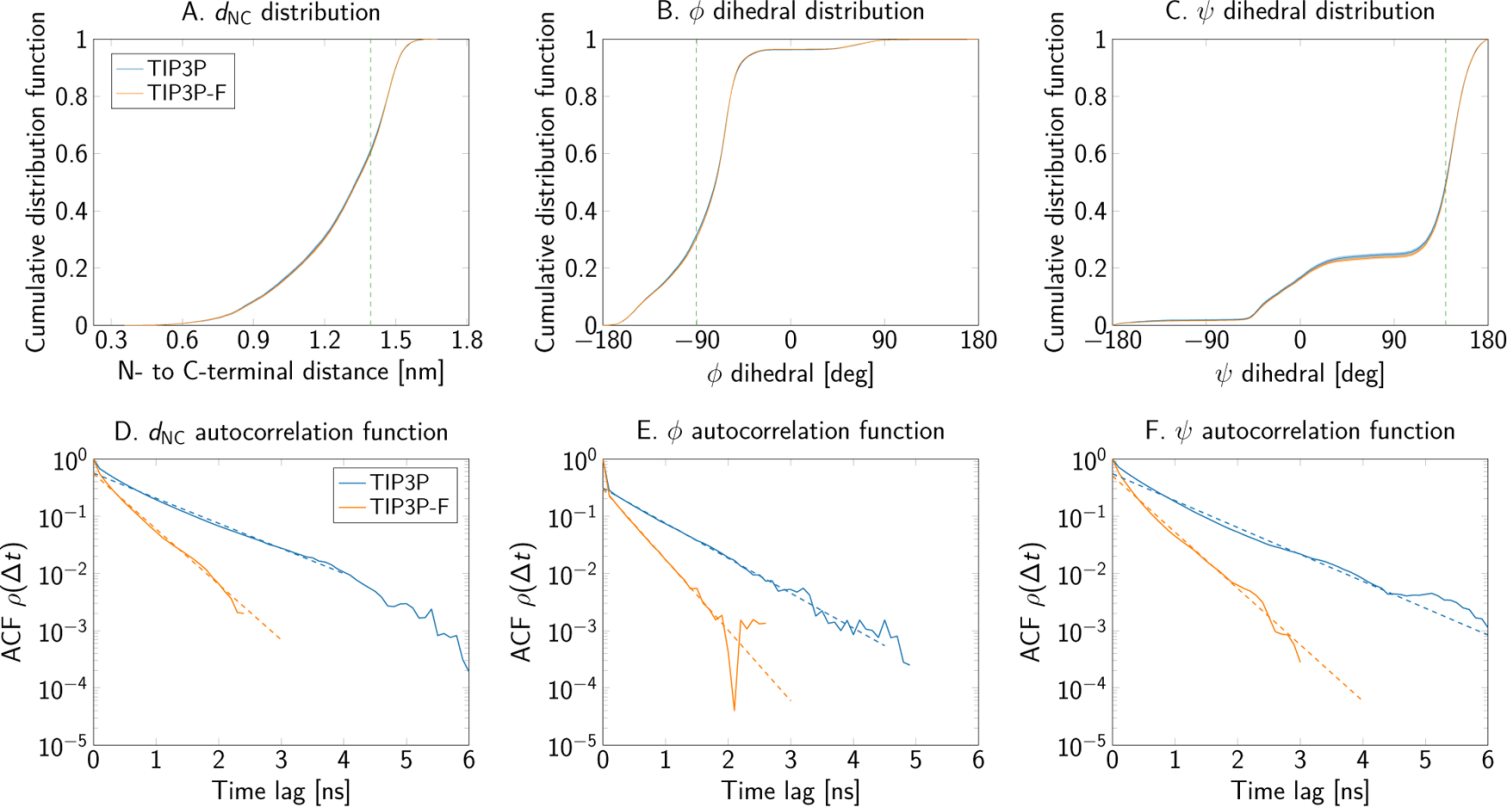
End-to-end distance and Ramachandran angles of the alanine pentapeptide as sampled in simulations using the TIP3P and TIP3P-F water models. (A–C) Cumulative distribution functions of the collective variables. The dashed vertical lines show where the two cumulative distributions are furthest apart. (D–F) Autocorrelation functions.

**Figure 10:**
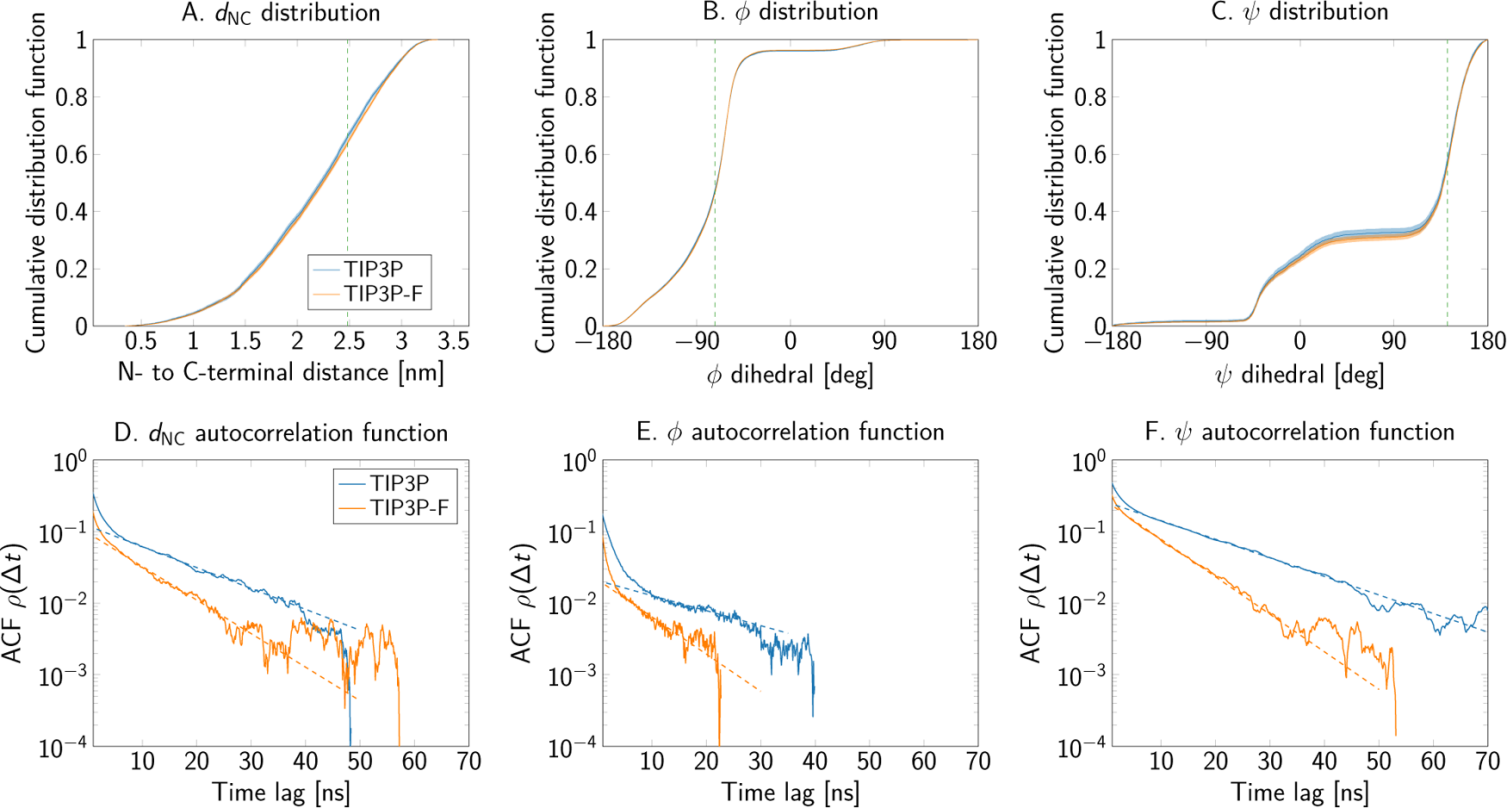
End-to-end distance and Ramachandran angles of the alanine decapeptide as sampled in simulations using the TIP3P and TIP3P-F water models. (A–C) Cumulative distribution functions of the collective variables. The dashed vertical lines show where the two cumulative distributions are furthest apart. (D–F) Autocorrelation functions.

Similar results were obtained for alanine penta- and decapeptides. Figures 9A-C and 10A-C depict the comparison of the cumulative distribution functions of the collective variables, sampled from simulations with each water model. Expectation values and order parameters of these observables are reported in Table 4. Small shifts toward larger values of the end-to-end distance were obtained with the mass-scaled TIP3P-F model. However, the magnitude of these differences is around 0.01 nm, and thus negligible for most applications. Furthermore, free energy difference maps of the Ramachandran angles of the central residue, presented in Figures S3 and S4, also demonstrate that the largest deviations are observed only at the edges of the map.

We determined whether the samples of the collective variables obtained using TIP3P and TIP3P-F come from the same equilibrium distribution by performing Kolmogorov–Smirnov (KS) tests. Since direct application of the KS test is complicated by the presence of correlations, numerical distributions of the statistic itself were calculated by comparing the samples from individual molecular dynamics trajectories. Results are presented in Figures S8–S12. The distribution of the values of the KS statistic when comparing trajectories simulated with different water models largely overlap with the distributions calculated when comparing trajectories with the same water model. These results evidence that the sampled equilibrium distribution is not altered substantially by the changes in the mass of the water molecule. It is also important to note that KS values from the simulations with the TIP3P-F model tend to be smaller than those observed between trajectories simulated with the standard TIP3P model. This means that the more efficient sampling by using the TIP3P-F model also results in less variability when comparing trajectories from independent molecular dynamics runs. We note, however, that the differences in the mean end-to-end distances and order parameters, while small in absolute terms, are in some cases statistically significant.

The scaling and repartition of the mass in the water molecules has large effects on the autocorrelation functions of the collective variables. Figures 8E,F, 9D–F, and 10D–F present the autocorrelation functions of each order parameter and collective variable of the peptide systems. From these plots, we conclude that the decrease in water viscosity due to the mass scaling scheme indeed leads to a faster decorrelation. Furthermore, the integrated autocorrelation times drop by about a factor of 2 for most systems (Table 4). Additionally, the exponential autocorrelation time, τ̂_exp_ was also calculated by linear regression over a reasonably linear regime in log space for the slowest observed process in the autocorrelation function. Dashed lines in the autocorrelation plots of Figures 8 to 10 show the range of the function used for the fits. In general, the amount of decrease in τ̂_exp_ is consistent with the observed decrease of the integrated times.

In molecular dynamics simulations, the desired accuracy of an estimator is limited by the integrated autocorrelation time τ_int_ because the statistical error decreases roughly as the variance times (2τ_int_/*t*)^1/2^ with the simulation time *t*.^43^ From the results above, this implies that simulations with the TIP3P-F model can result in smaller estimation errors for the same simulation lengths. Equivalently, “independent” samples can be generated within fewer molecular dynamics steps. Indeed, the error bars calculated here with block averages (which do not require the autocorrelation time explicitly), tend to be smaller for the systems simulated with the TIP3P-F model over the same total time. Furthermore, Figures S5 to S7 show the cumulative distribution function of all collective variables calculated from the alanine decapeptide simulations at different points in time. Clearly, the time evolution of the CDFs with the TIP3P model leads to a notably smaller uncertainty for the same simulation length.

**Figure 11:**
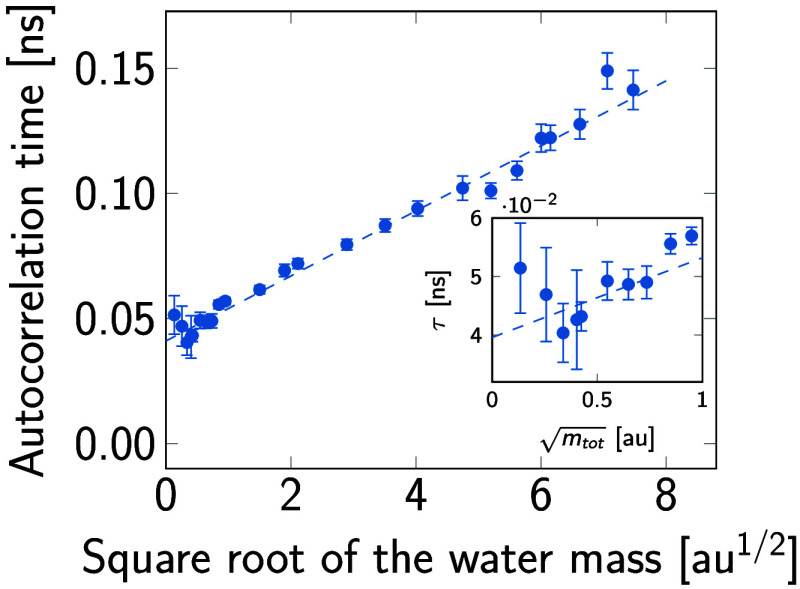
Dependence of the alanine dipeptide ψ integrated dihedral autocorrelation time, τ_int_, on the square root of the mass of the water molecules, . The repartitioned mass *m*_r_ = 4 of the TIP3P-F model was kept fixed in the simulations. The inset shows the estimated autocorrelation times for the smallest water masses tested in this work.

Interestingly, the acceleration in the relaxation times is somewhat less than the roughly 3-fold change in solvent self-diffusion or viscosity. For the solutes, the autocorrelation times instead decrease by about a factor of 2. To explore this effect, we performed molecular dynamics simulations of the alanine dipeptide by scanning through different total masses of the water molecules, using τ_int_ of the ψ dihedral as a reporter variable. The results in Figure 11 show that autocorrelation times are approximately proportional to the square root of the water mass, as should be expected according to eq 3, and as seen before for peptides.^44^ Moreover, a least-squares straight-line fit with respect to the square root of the mass scale factor (Table 5) reveals that the solute autocorrelation time does not go to zero after extrapolation to zero mass. A small upturn of τ_int_ as *m*_tot_ → 0 is not statistically significant, but would be consistent with a transition into the Kramers low-friction regime,^9,12^ where the exchange of energy between solute and solvent degrees of freedom becomes inefficient. However, much shorter time steps would be required for a firmer assessment of this interesting regime.^44^

**Table 5:** Fit of  to the Integrated Autocorrelation times of the ψ Dihedral Angle of Alanine Dipeptide as a Function of Total Water Mass (See Figure 11)^a^

parameter	fitted value
*a* [ns]	0.041 (0.001)
*b* [ns × au^–1/2^]	0.013 (0.0003)

a Uncertainties correspond to the standard errors of the slope and intercept.

From a mechanistic point of view, solute motion is affected by forces arising from solute–solvent and solute–solute interactions. Conformational changes in the peptide that require large motions or rearrangements of water networks are limited by the time needed for solvent reorganization, that is, the solvent exerts friction over the solute. The proportionality constant of the model in Table 5, *b*, is a measure of the strength of solute–solvent friction. Increasing the water diffusion constant decreases the time for solvent reorganization. Therefore, as the water mass approaches zero, the dynamics of solute degrees of freedom is dominated by a combination of intramolecular and thermostat friction. This “residual” autocorrelation time is characterized by the *a* parameter in Table 5, which quantifies the “internal friction”^12,44,45^ associated with couplings to intramolecular motions combined with thermostat friction. In practical terms, *a* represents a limit in the maximum speed-up that can be achieved by scaling the solvent mass. This theoretical limit is well within the bounds of integration stability of the TIP3P-F model and provides justification for our final water mass. As Figure 11 shows, by using a total mass, *m*_tot_ = 1.116 g/mol, we are already within the error bar of the theoretical limit of the alanine dipeptide system.

### 3.4 Validation and Sampling Efficiency for Nucleic Acids

To test if the increase in sampling efficiency observed in protein dynamics extends to other biomolecules, we prepared systems of the polyribonucleotides Ade_2_ and Ade_4_ with both water models. In contrast to polypeptides, where backbone dynamics can be characterized by the two Ramachandran dihedrals, nucleic acids have six different torsion angles shared between consecutive residues. Here, we only analyzed the phosphate α and ζ dihedrals of the central residues, together with the distance between the O5′ and the O3′ atoms of terminal ribonucleotides (*d*_O5′–O3′_). Figure 7 shows the structures and variables used for the polyribonucleotides.

**Figure 12:**
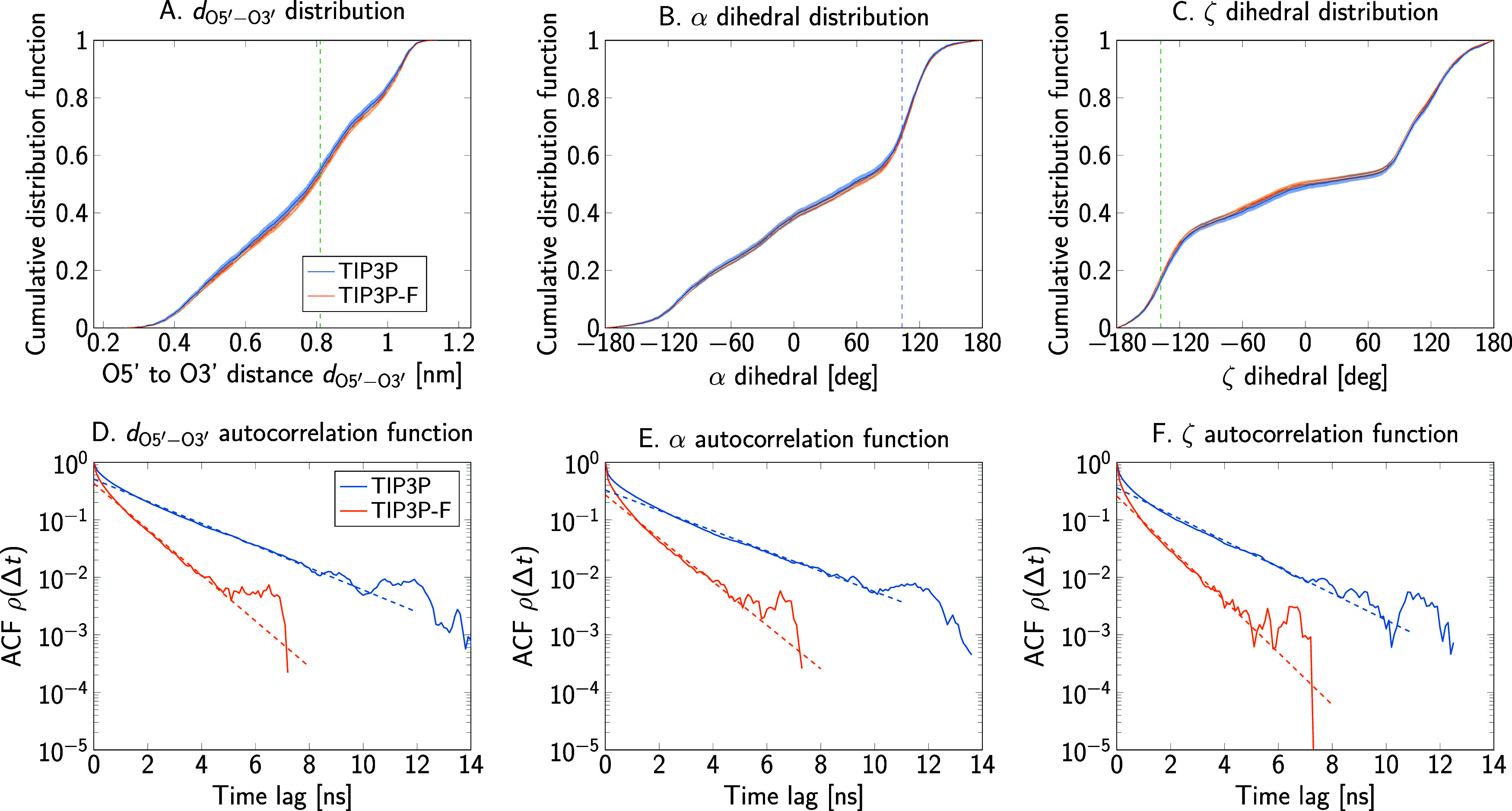
End-to-end distance and dihedral angles of the adenine diribonucleotide as sampled in simulations using the TIP3P and TIP3P-F water models. (A–C) Cumulative distribution functions for the O5′ to O3′ distance between terminal nucleotides and for the phosphate bound α and ζ dihedrals. The dashed vertical lines show where the two cumulative distributions are furthest apart. (D–F) Autocorrelation functions.

**Figure 13:**
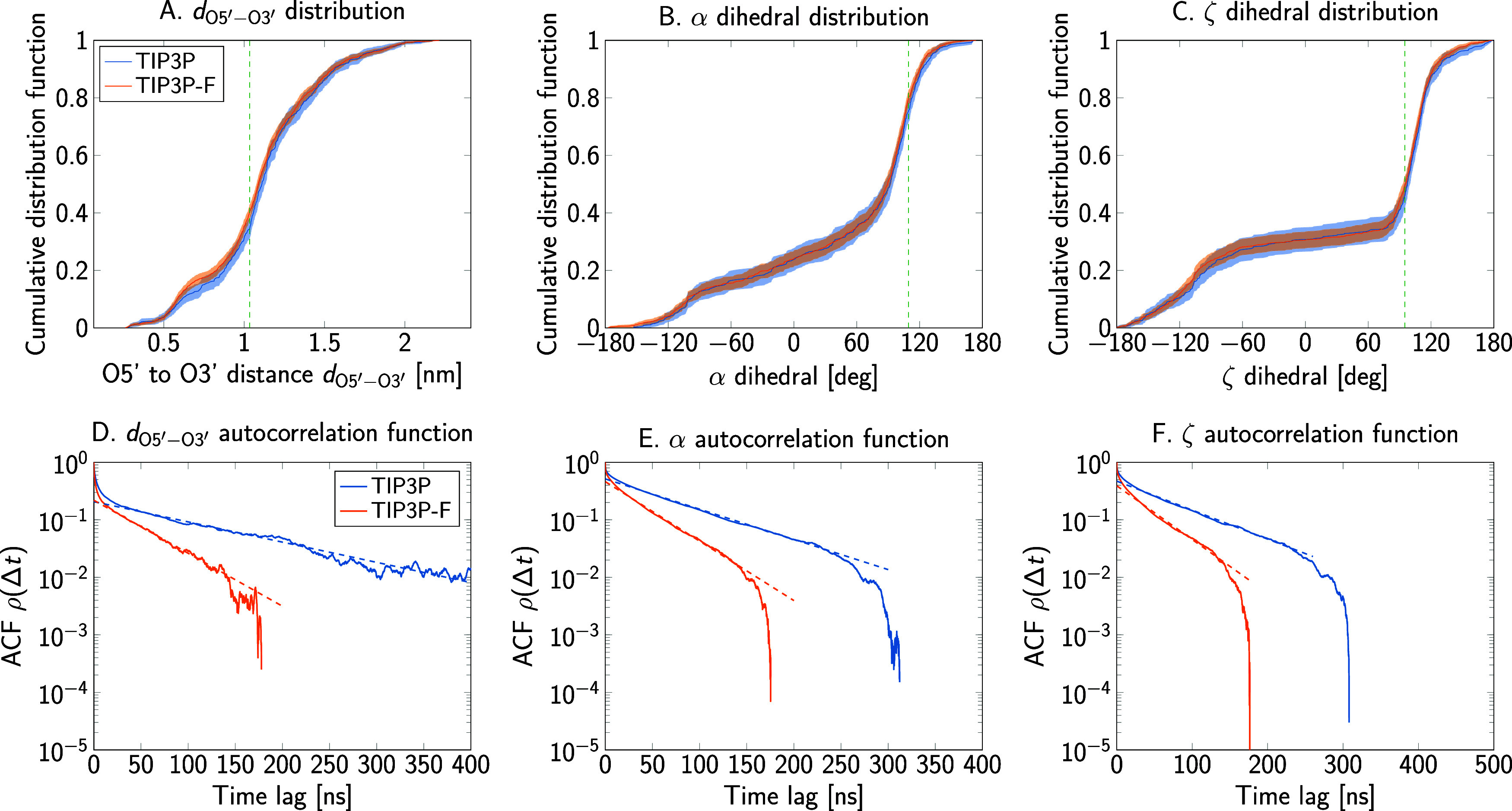
End-to-end distance and dihedral angles of the adenine tetraribonucleotide as sampled in simulations using the TIP3P and TIP3P-F water models. (A–C) Cumulative distribution functions for the O5′ to O3′ distance between terminal nucleotides and for the phosphate bound α and ζ dihedrals. The dashed vertical lines show where the two cumulative distributions are furthest apart. (D–F) Autocorrelation functions.

As for the peptide observables, the calculated cumulative distribution function of *d*_O5′–O3′_ is practically unchanged when performing simulations with TIP3P-F (Figures 12A and 13A). Accordingly, the free energy profile can be completely recovered with small differences mainly due to noise. As shown in Table 6, the averages of this distance also coincide within the calculated error. Values of the KS statistic for the *d*_O5′–O3′_ distributions sampled with the different water models have similar magnitude, as shown in Figure S13. This behavior is also observed for the phosphate dihedrals, shown in Figures 12B,C and 13B,C, with corresponding KS statistics shown in Figures S14–S16. Cumulative distribution functions and their associated dihedral order parameters are practically independent of the water model and the estimated errors are within expected fluctuations between independent molecular dynamics runs.

**Table 6:** Dihedral Order Parameters and Expectation Values of Collective Variables Used to Analyze the Simulations of the Polyribonucleotide Systems

RNA	collective variable	water model	average (SE)	τ̂_int_ (SE) [ns]	τ̂_exp_ (SE) [ns]
Ade_2_	*d*_O5′–O3′_ [nm]	TIP3P	0.760 (0.002)	1.270 (0.047)	2.254 (0.015)
		TIP3P-F	0.759 (0.002)	0.561 (0.015)	1.090 (0.013)
	*S*_D_^2^(α)	TIP3P	0.046 (0.003)	0.992 (0.036)	2.460 (0.026)
		TIP3P-F	0.045 (0.002)	0.428 (0.011)	1.148 (0.019)
	*S*_D_^2^(ζ)	TIP3P	0.105 (0.002)	0.797 (0.028)	1.889 (0.021)
		TIP3P-F	0.107 (0.003)	0.345 (0.009)	0.957 (0.016)
Ade_4_	*d*O5′–O3′ [nm]	TIP3P	1.113 (0.010)	28.321 (3.433)	122.075 (0.420)
		TIP3P-F	1.109 (0.006)	11.258 (0.802)	47.004 (0.124)
	*S*_D_^2^(α)	TIP3P	0.263 (0.017)	42.128 (3.979)	82.005 (0.104)
		TIP3P-F	0.288 (0.012)	20.098 (1.423)	42.004 (0.064)
	*S*_D_^2^(ζ)	TIP3P	0.191 (0.013)	40.492 (3.799)	85.540 (0.107)
		TIP3P-F	0.212 (0.008)	19.332 (1.373)	46.473 (0.116)

Autocorrelation functions of the end-to-end distance in Figures 12D and 13D demonstrate that faster convergence can be achieved with TIP3P-F. Comparison of τ_int_ indicates a roughly 2-fold increase in efficiency, comparable to the case of the alanine polypeptides. The relaxation time τ_exp_ corresponding to the slowest, exponentially decaying process was evaluated by linear regression. Results for τ_exp_ are presented alongside τ_int_ in Table 6, and the fits are shown as dashed lines in the autocorrelation plots of Figures 12D and 13D. Moreover, the increase in the rate of convergence is also observed for other variables, namely the α and ζ dihedrals, as demonstrated by the autocorrelation functions in Figures 12E,F and 13E,F.

### 3.5 Validation and Sampling Efficiency for Protein

**Figure 14:**
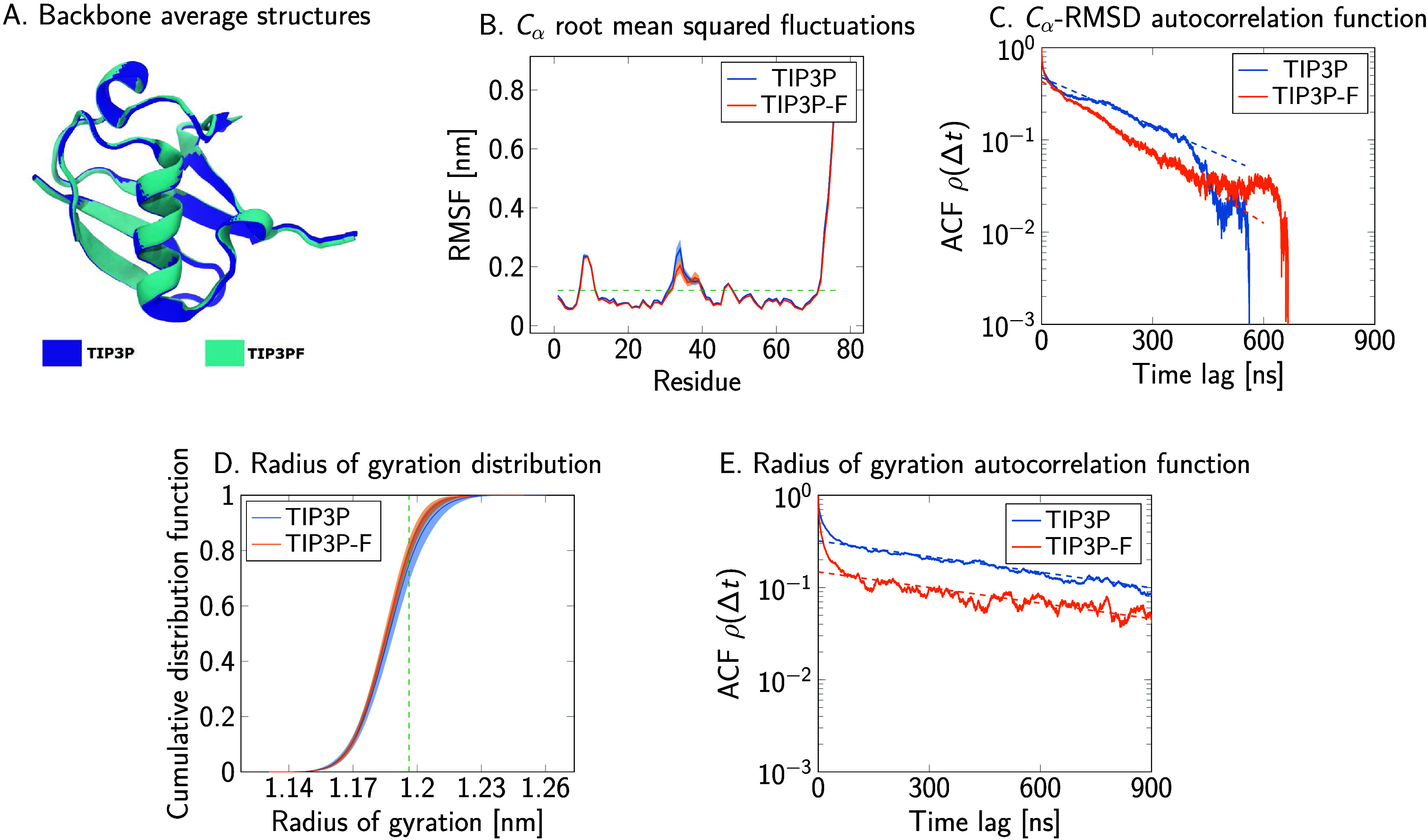
Superposition of the average backbone structure generated from trajectories using TIP3P and TIP3P-F water models (A). (B, C) Root-mean-square fluctuation (RMSF) values of the α carbons per residue, and the autocorrelation function of the root-mean-square deviation (RMSD) with respect to the average structures, respectively. The horizontal dashed line in the RMSF plot shows the cutoff used to define the ubiquitin core. Empirical cumulative distribution functions of the radius of gyration of ubiquitin (D) comparing the sampling using the TIP3P and TIP3P-F water models. The dashed vertical lines show where the two cumulative distributions are furthest apart. Autocorrelation functions of the time series are presented in (E). Dashed lines indicate linear regression fits of the late decay of the slowest observed process.

Results discussed in previous sections showed a roughly 2-fold increase in sampling efficiency using the TIP3P-F water model for small peptide and ribonucleotide systems. To test if similar effects can be expected in simulations of properly folded proteins, we selected ubiquitin^46^ as a model system. In total, 10 independent molecular dynamics simulations of ubiquitin with a length of 2.7 μs per replica were used to calculate statistics and for error analysis. First, time series of the radius of gyration, *R*_g_, were generated to calculate their distribution and expectation value. Figure 14D compares the numerical cumulative distribution functions observed in simulations with TIP3P and TIP3P-F. The empirical distributions and the averages in Table 7 indicate that simulations with both water models lead to essentially the same results. Additionally, Figure 14E shows a substantial decrease in the decorrelation rate of this variable when using the TIP3P-F water model, particularly in the short- and mid-time scale. The amount of acceleration, as assessed quantitatively by comparing the integrated autocorrelation times of both systems in Table 7, is consistent with previous results. On the other hand, the exponential characteristic time of the slowest apparent process is not clearly different between the two water models. The associated motions may thus be dominated by “internal friction”. However, the signal-to-noise ratio in the points used for the linear fit is low, making differences in the slope difficult to detect, and the respective time scale approaches the overall duration of the simulations.

**Table 7:** Ensemble Averages and Autocorrelation Analysis of the Radius of Gyration of Ubiquitin from Molecular Dynamics Simulations Data

water model	average (SE) [nm]	τ̂_int_ (SE) [ns]	τ̂_exp_ (SE) [ns]
TIP3P	1.187 (0.001)	191.4 (85.3)	716.30 (1.28)
TIP3P-F	1.185 (0.001)	99.1 (46.6)	724.61 (3.57)

In addition to the *R*_g_ time series, the collective dynamics of the α-carbon backbone atoms was analyzed. First, an average protein structure in TIP3P and TIP3P-F water was calculated by repeatedly aligning and averaging all structures from the respective molecular dynamics runs until convergence was achieved. Figure 14A shows a superposition of the average structures from trajectories using the two water models, demonstrating that the average backbone conformation is independent of water mass. Afterward, the root-mean-square fluctuation (RMSF) values of every α carbon atom about their average position were generated and a cutoff of 0.1 nm was applied to select a rigid skeleton for the generation of the time series, as shown in Figure 14B. Then, the trajectories were realigned with respect to this ubiquitin core. Time series of the RMSD of the α carbon atoms from the core average were generated. The autocorrelation functions of the time series are depicted in Figure 14C and the integrated and exponential autocorrelation times are reported in Table 8. According to the results, the TIP3P-F water model reduces the decorrelation times by about 30 to 40% with respect to the standard TIP3P model. Therefore, the TIP3P-F model speeds up the dynamics also for a folded protein. However, the slowest motions captured in the autocorrelation functions appear to decay with similar characteristic times τ_exp_ for TIP3P and TIP3P-F, indicating that the underlying processes are dominated by internal friction.

**Table 8:** Autocorrelation times of the Core-RMSD Time Series for the Simulations of Ubiquitin

water model	τ̂_int_ (SE) [ns]	τ̂_exp_ (SE) [ns]
TIP3P	100 (29)	248
TIP3P-F	78 (27)	169

### 3.6 Validation and Sampling Efficiency for Lipid Bilayer

**Figure 15:**
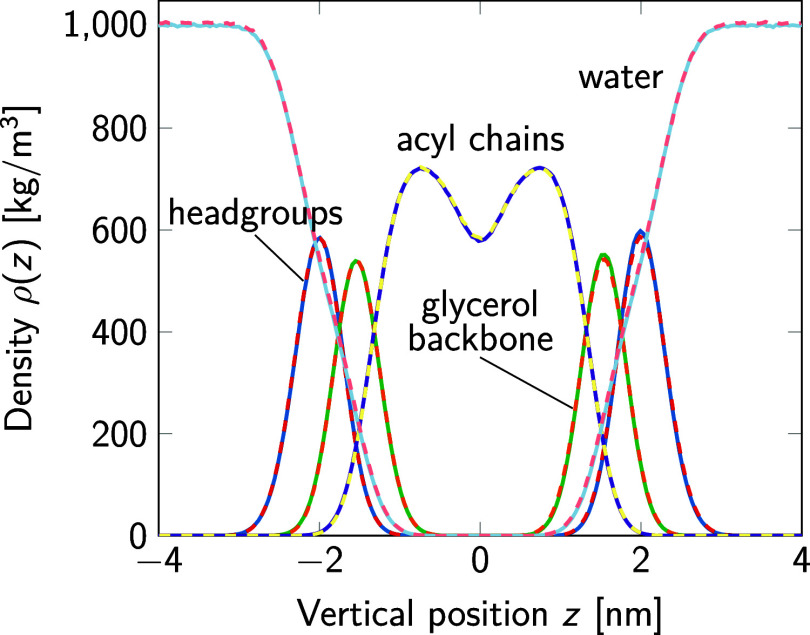
Mass density profiles of the lipid headgroups, glycerol backbone, acyl chains, and water in simulations of POPC bilayers using the TIP3P (solid lines) and TIP3P-F (dashed lines) water models. For the analysis of the water mass density, standard water masses were used in both cases.

**Figure 16:**
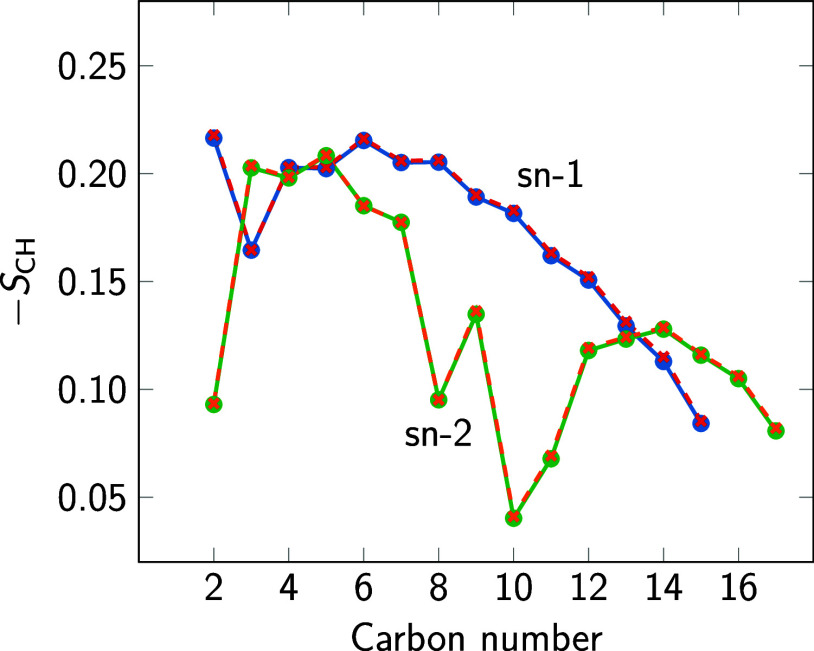
*S*_CH_ order parameter of the sn-1 and sn-2 acyl chains calculated in simulations of POPC bilayers using the TIP3P (solid lines as a guide to the eye) and TIP3P-F (dashed lines) water models.

Figures 15 and 16 indicate that NPT simulations with the TIP3P and TIP3P-F leave the density and order parameter of the lipid bilayer virtually unchanged, in agreement with the expectation that equilibrium thermodynamic averages are independent of the mass distribution of the system. Moreover, Table 9 shows that the area per lipid values are also identical across the two water models. However, due to the reduced viscosity of the surrounding aqueous medium, the apparent diffusion coefficient of the lipids, without finite-size correction,^35,47^ is increased by ∼25%.

**Table 9:** Comparison of Area Per Lipid (APL) and Lipid Diffusion Coefficient between Simulations of POPC Using TIP3P and TIP3P-F^a^

water model	APL (SE) [Å^2^]	*D* (SE) [10^–3^ nm^2^/ns]
TIP3P	65.0 (0.1)	9.9 (0.6)
TIP3P-F	65.1 (0.1)	12.4 (0.3)

a Uncertainties report the difference between the two replicas.

The enhanced sampling brought about by the decreased viscosity of the TIP3P-F model is less than what we observed for small biomolecules. The reason for this is that lipid mobility is dominated by the membrane viscosity.^35,47,48^ To speed up the lipid dynamics more substantially, one could use mass repartitioning and scaling also for lipids.

## 4 Conclusions

We introduced the TIP3P-F model for fast sampling of equilibrium distributions of (bio)molecules in aqueous solution. By first mass repartitioning mass from the heavy oxygen to the light hydrogen atoms and then rescaling the mass of the entire water molecule, we achieved a roughly 3-fold decrease in solvent viscosity while maintaining the time integration stability. By changing only the masses, TIP3P-F can be used with all force fields compatible with TIP3P water, with the usual time step of Δ*t* ≈ 2 fs.

We demonstrated that decreasing solvent viscosity enhances conformational sampling in biomolecular simulations. Substantial speedups in sampling by about a factor of 2 for solvated biomolecules, proteins, and nucleic acids, and by ∼25% for fully hydrated lipid bilayers afforded by the TIP3P-F model are achieved without compromising the structural and thermodynamic properties of solute and solvent.

We would be remiss not to emphasize that the TIP3P-F water model presented here can be trivially combined with the conventional HMR schemes that allow 4 fs time steps. When using TIP3P-F in HMR simulations with *doubled*, 4 fs time step, the total water mass *m*_tot_ of TIP3P-F should be *quadrupled* to *m*_tot_^HMR^ = 4.464 so not to alter the crash rate *k*_crash_ of the system. We refer to the literature^4,5^ for guidance on HMR.

For practitioners, if retaining the actual dynamics is a concern, TIP3P-F can be used only in the equilibration phase to create well-sampled starting points for runs with regular TIP3P. If sampling is the focus, as is usually the case, TIP3P-F can be used also for the production runs. The use of TIP3P-F should also further accelerate enhanced sampling methods. In umbrella sampling, for instance, sampling will speed up by a factor given by the decrease in the decorrelation time in the windows as a result of the faster solvent motions.^49^ However, for calculations requiring high precision, as in certain tests on force fields, one may want to increase the total mass *m*_tot_ in the TIP3P-F model to a value intermediate between 1.116 of TIP3P-F and 18.0154 of TIP3P (Table 1), trading off statistical against systematic errors. Overall, we recommend TIP3P-F for the efficient sampling of (bio)molecular systems, resulting in roughly 2-fold speed-ups in time-to-discovery and, with that, ∼50% reductions in energy cost and reduced climate impact.^50^

## Supporting Information Available

Detailed description of all simulations (Table S1); fit parameters used in the crash rate equation (Table S2); kinetic energy partitioning (Figure S1); difference in free energy for alanine dipeptide Ramachandran map (Figure S2); difference in free energy for alanine pentapeptide Ramachandran map (Figure S3); difference in free energy for alanine decapeptide Ramachandran map (Figure S4); convergence of end-to-end distance of alanine decapeptide (Figure S5); convergence of central ϕ dihedral angle in alanine decapeptide (Figure S6); convergence of central ψ dihedral angle of alanine decapeptide (Figure S7); KS statistics for ϕ and ψ dihedrals in alanine dipeptide (Figure S8); end-to-end distance of alanine pentapeptide (Figure S9); central Ramachandran angles in alanine pentapeptide (Figure S10); end-to-end distance of alanine decapeptide (Figure S11); central Ramachandran dihedrals in alanine decapeptide (Figure S12); end-to-end distance of adenine diribonucleotide (Figure S13); central phosphate dihedrals in adenine diribonucleotide (Figure S14), end-to-end distance of adenine tetraribonucleotide (Figure S15); and central phosphate dihedrals in adenine tetraribonucleotide (Figure S16) (PDF)
