# Supplementary material for: Faster Sampling in Molecular Dynamics Simulations with TIP3P-F Water
Source: J Chem Theory Comput. 2024 Dec 12;20(24):11068–81. doi: 10.1021/acs.jctc.4c00990 (PMC11672673; doi:10.1021/acs.jctc.4c00990)
Supplement: Supplementary file 1 — ct4c00990_si_001.pdf [file ct4c00990_si_001.pdf]

Supporting Information: Faster Sampling in Molecular Dynamics Simulations with TIP3P-F Water

José Guadalupe Rosas Jiménez,[†,‡,§] Balázs Fábián,[†,§] and Gerhard Hummer[*,†,¶]

†*Department of Theoretical Biophysics, Max Planck Institute of Biophysics, Max-von-Laue-Straße 3, 60438 Frankfurt am Main, Germany*

‡*IMPRS on Cellular Biophysics, Max Planck Institute of Biophysics, Max-von-Laue-Straße 3, 60438 Frankfurt am Main, Germany*

¶*Institute of Biophysics, Goethe University Frankfurt, 60438 Frankfurt am Main, Germany*

§*These authors contributed equally to this work.*

* E-mail: gerhard.hummer@biophys.mpg.de

Contents

1 Supporting Text **S2**

1.1 Autocorrelation Analysis S2

1.2 Comparison of Equilibrium Distributions S3

1.3 Numerical Distributions of the Kolmogorov-Smirnov Statistic S4

2 Supporting Tables S1–S2 **S6**

3 Supporting Figures S1–S16 **S7**

References **S18**

1 Supporting Text

1.1 Autocorrelation Analysis

The sampling rate and efficiency were assessed by means of autocorrelation analysis of selected collective variables in the model systems. First, the autocorrelation, $C(t)$, and normalized autocorrelation functions, $\rho(t)$, were estimated using eqs 1 and 2, where carets ($\wedge$) denote the estimator of a quantity and $\bar{f}$ is the average value of the collective variable:

$$\hat{C}(t) = \frac{1}{n-t} \sum_{i=1}^{n-t} \left[f(t_i) - \bar{f} \right] \left[f(t_i + t) - \bar{f} \right] \tag{1}$$

$$\hat{\rho}(t) = \hat{C}(t) / \hat{C}(0) \tag{2}$$

Collective variable averages $\bar{f}$ were calculated over all repetitions. $\hat{C}(\delta t)$ was calculated by averaging over all runs and then normalized over the variance from all repetitions. Then, the integrated autocorrelation time, τ_{int}, is calculated numerically using the trapezoid rule as implemented in SciPy.[S1]

Since the variance of $\hat{\tau}_{\text{int}}$ grows at long lag times due to accumulation of errors, a cutoff, M, must be introduced in its calculation. Here, we truncate the summation at the point where the normalized autocorrelation function crosses zero. Following the derivation by Sokal,[S2] given that $\tau_{\text{int}}/\delta t \ll M \ll n$, the variance in the estimation of $\hat{\tau}_{\text{int}}$ can be approximated by:

$$\text{var}\left(\hat{\tau}_{\text{int}}\right) \approx \frac{2\left(2M+1\right)}{n} \tau_{\text{int}}^2 \tag{3}$$

From the value of the integrated autocorrelation time, the effective standard error of the estimation of f can then be calculated as:

$$\text{SE}(\hat{f}) = \sqrt{\text{var}(\hat{f})} \approx \sqrt{\frac{1}{n}\left(2\tau_{\text{int}}\right) C(0)} \tag{4}$$

given that $n \gg \tau_{\mathrm{int}}$.

For the specific case of dihedral angle time series, we used the definition of the dihedral autocorrelation function by van der Spoel and Berendsen:[S3]

$$C_{\mathrm{D}}(\delta t) = \langle P_1(\cos[\theta(t) - \theta(t+\delta t)])\rangle_t = \langle \cos[\theta(t) - \theta(t+\delta t)]\rangle_t \,, \tag{5}$$

where $P_1(x) = x$ is the first Legendre polynomial, t is any starting time and δt is the lag-time. Equation 5 can also be written as a sum of two products:

$$\begin{aligned} C_{\mathrm{D}}(\delta t) = \langle \cos[\theta(t)] \cos[\theta(t+\delta t)]\rangle_t \\ + \langle \sin[\theta(t)] \sin[\theta(t+\delta t)]\rangle_t \end{aligned} \tag{6}$$

After normalization, integrated autocorrelation times were calculated as explained above. At long times, $C_{\mathrm{D}}(t)$ converges to the expression:

$$S^2_{\mathrm{D}}(\theta) \equiv C_{\mathrm{D}}(\delta t \to \infty) = \langle \cos\theta\rangle^2 + \langle \sin\theta\rangle^2 \tag{7}$$

where S^2_{D} is the dihedral order parameter, characteristic of the equilibrium distribution of the dihedral values. Since S^2_{D} is a sum of squared averages, the uncertainty in its estimation cannot be calculated directly from eq 4. The statistical error in this parameter was determined using block average analysis, plotting the estimated standard error as a function of the block length until a plateau is reached.

1.2 Comparison of Equilibrium Distributions

To demonstrate that changes in water mass have a negligible effect on equilibrium distribution of biological systems, the cumulative distribution function (CDF) of selected collective variables was estimated from molecular dynamics simulations data. The CDF estimator,

$\hat{F}(x)$, is defined as:

$$\hat{F}(x) = \frac{1}{n}\sum_{i=1}^{n} \mathbf{1}_{(-\infty,x)}\left[x_i\right] \tag{8}$$

where $\mathbf{1}_{(-\infty,x)}\left[x_i\right]$ is the indicator function with a value of one if $x_i < x$ and zero otherwise. Using this definition of the CDF estimator, error bars were calculated using block averages, as described by Bussi and Tribello.[S4]

Finally, the Kolmogorov-Smirnov (KS) statistic, implemented in SciPy,[S1] and its numerical distribution between independent molecular dynamics runs were used to quantify the difference between the CDF of collective variables measured in simulations using TIP3P and TIP3P-F.

1.3 Numerical Distributions of the Kolmogorov-Smirnov Statistic

In the two-sample Kolmogorov-Smirnov test, the distance between two empirical cumulative distribution functions is measured as the largest absolute difference between observed values:

$$D_{n,m} = \sup\left|\hat{F}_{a,n}(x) - \hat{F}_{b,m}(x)\right| \tag{9}$$

$\hat{F}_{a,n}(x)$ and $\hat{F}_{b,m}(x)$ are the estimators of the CDFs for distributions a and b from samples of size n and m, respectively. $D_{n,m}$ follows a Kolmogorov distribution whose parameters are the sample sizes, n and m. However, here the usual assumption of independent and identically distributed samples is violated. In molecular dynamics simulations, observations are independent only when sampled at time intervals longer than the decorrelation time. Therefore, the size of independent samples will be smaller than the total number of data points recorded in the simulation. Furthermore, the specific number of independent samples is difficult to accurately determine. For these reasons, a direct application of the analytical Kolmogorov distribution for the computation of p values is not possible.

In Figures S8 - S16, we instead show the numerical distributions of the KS statistic of the CDFs calculated from time series of collective variables for each pair of molecular dynamics

trajectories, comparing those within and between simulations with the TIP3P and TIP3P-F water models.

2 Supporting Tables

Table S1: Detailed description of all simulations discussed in this work

System	Number of water molecules	Simulation length per replica	Replicas
Water boxes	1024	100 ns	1
	2048	100 ns	1
	6800	100 ns	1
Ala-Dip	1722	4.0 µs	10
Ala_5	2492	3.0 µs	10
Ala_{10}	11280	5.0 µs	10
Ade_2	3628	4.0 µs	10
Ade_4	3785	15.0 µs	10
Ubiquitin	12281	2.7 µs	10
POPC membrane	11451	200 ns	2

Table S2: Fit parameters used in the crash rate equation, eq 6, in the main manuscript.

m_r	k^0_crash [$\mathrm{s}^{-1} \cdot 1000\ \mathrm{mols}^{-1}$]	c [au]
0	893.91	0.043
1	2.92	0.037
2	12.48	0.028
3	1.93	0.026
4	66.36	0.021
4.5	4.13	0.023
5	1.72	0.024
6	0.89	0.026
7	0.41	0.028
8	0.01	0.036

3 Supporting Figures

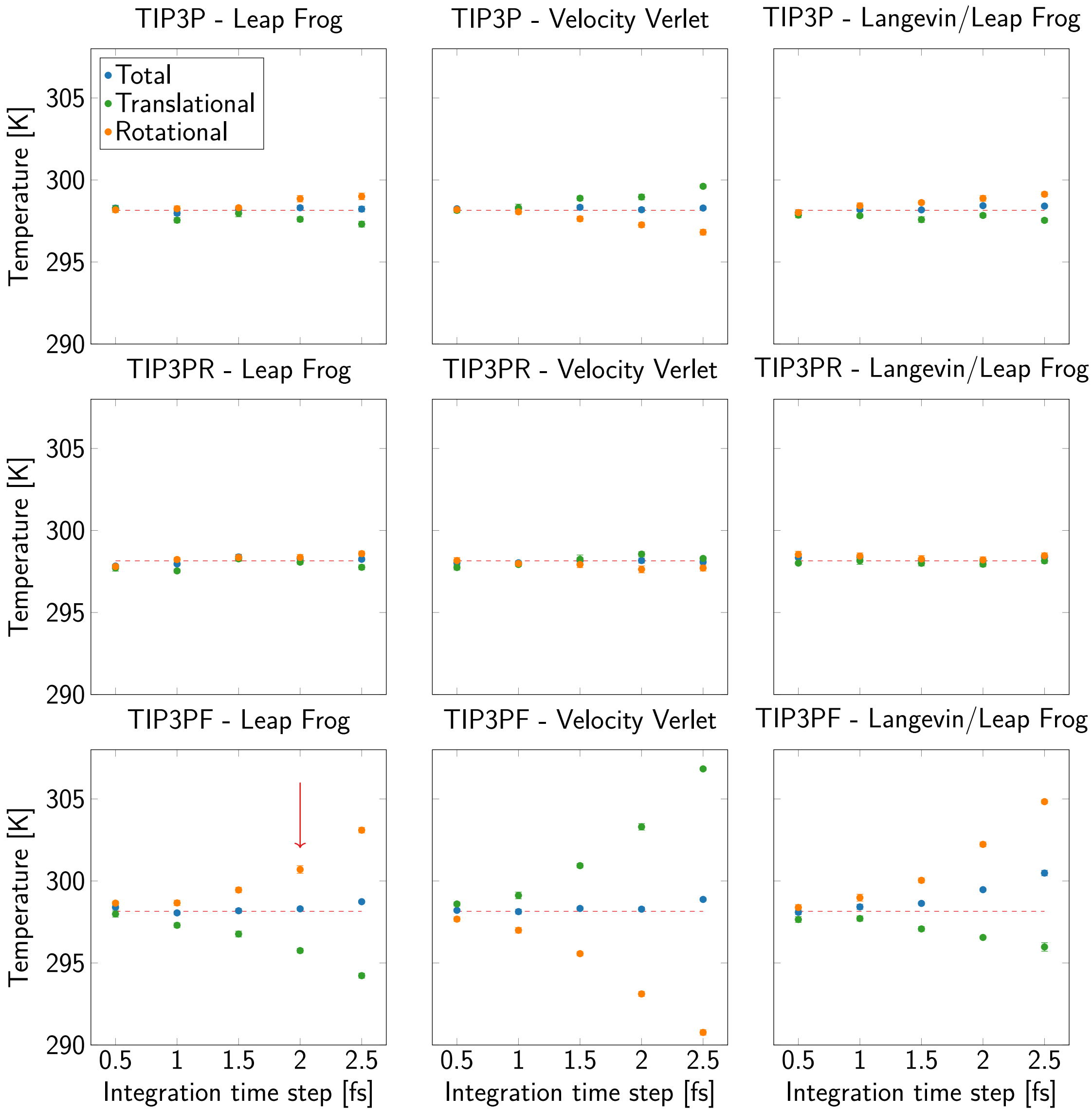

Figure S1: Analysis of the kinetic energy partitioning among rotational and translational degrees of freedom. The leap frog and velocity Verlet integrators were used in combination with the stochastic velocity rescaling thermostat (left and central panels). Results with the Langevin thermostat are presented in the right panels. TIP3P is the original base model, TIP3P-R is the model with hydrogen mass repartition, keeping the total mass, and TIP3P-F is the model with hydrogen mass repartition and mass scaling, as described in the main text. The horizontal, dashed line represents the target temperature. The arrow shows the simulation parameters were the TIP3P-F model was tested.

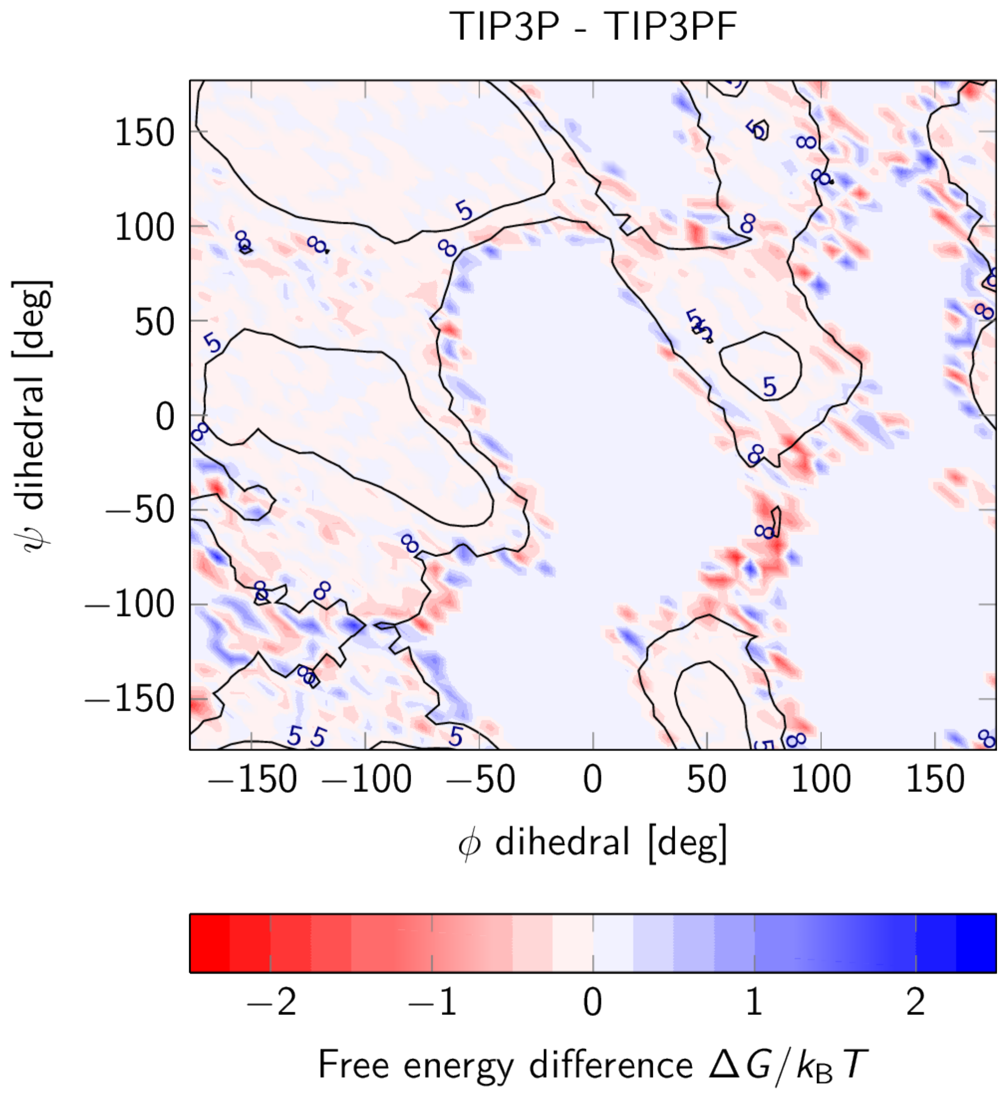

Figure S2: Difference in free energy calculated from the simulations of the alanine dipeptide, with respect to the TIP3P model results. Contour lines at $5k_BT$ and $8k_BT$ obtained with the original TIP3P model are shown as reference.

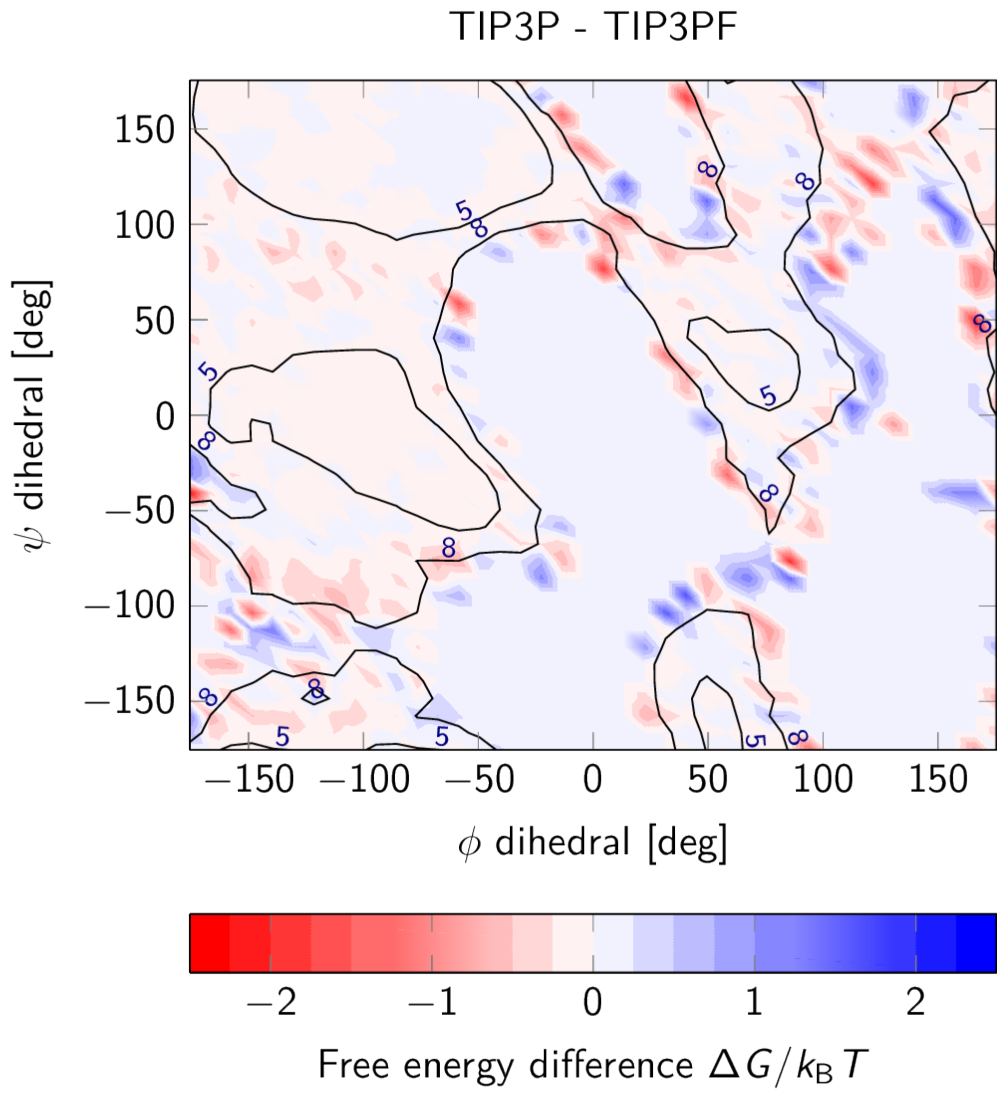

Figure S3: Difference in free energy calculated from the simulations of the alanine pentapeptide (central residue), with respect to the TIP3P model results. Contour lines at $5k_BT$ and $8k_BT$ obtained with the original TIP3P model are shown as reference.

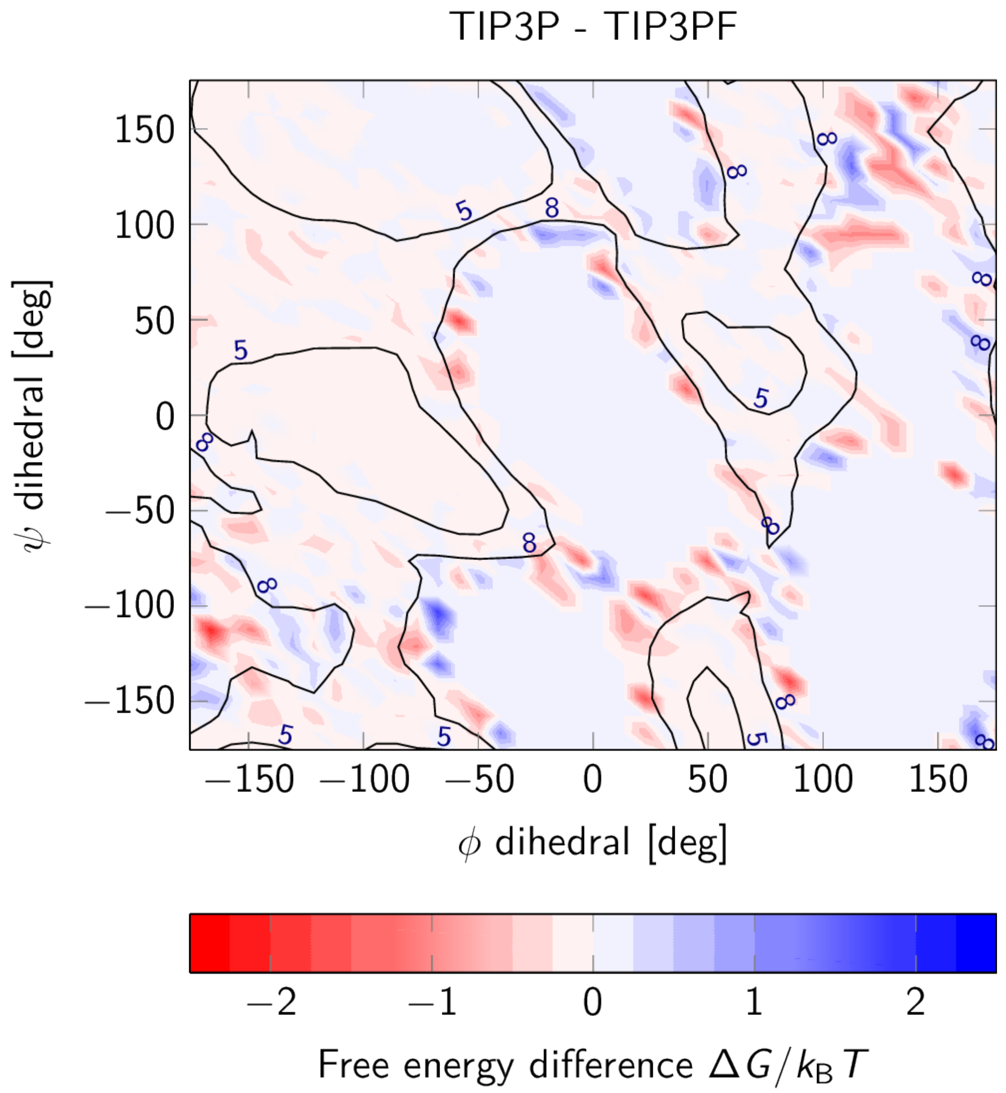

Figure S4: Difference in free energy calculated from the simulations of the alanine decapeptide (central residue), with respect to the TIP3P model results. Contour lines at $5k_BT$ and $8k_BT$ obtained with the original TIP3P model are shown as reference.

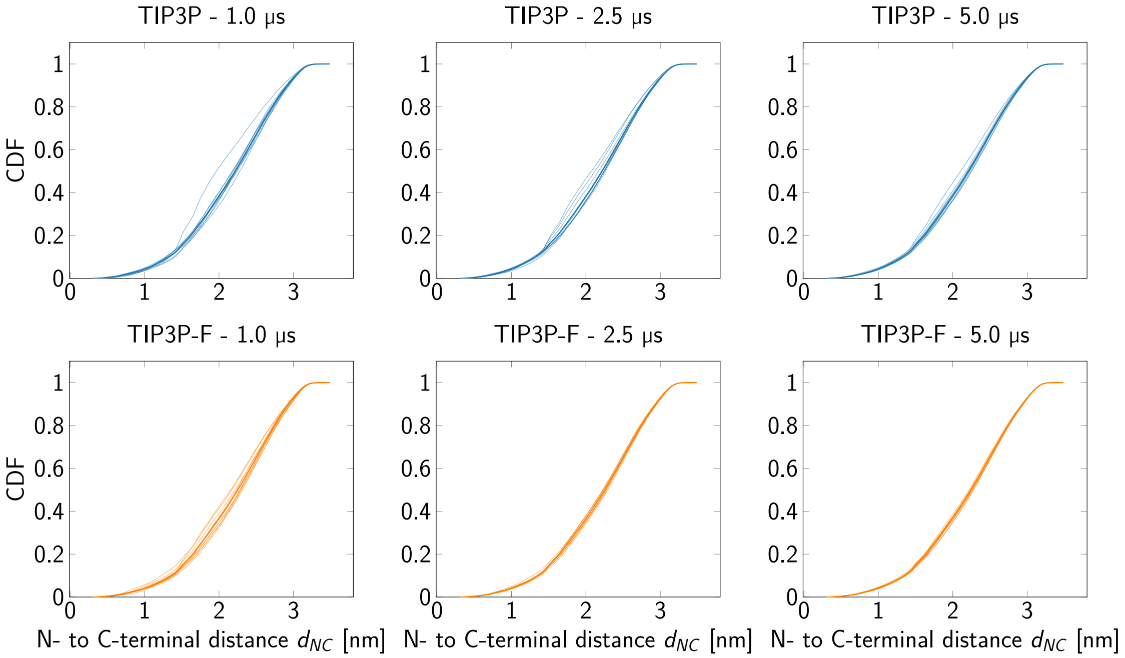

Figure S5: Convergence of the stationary distribution of the end-to-end distance of the alanine decapeptide. Panels show the cumulative distribution function up to different time points (left to right) in the respective simulations for TIP3P (top) and TIP3P-F water (bottom). Light and dark colored lines show the distributions for different replicates and the final average distribution, respectively.

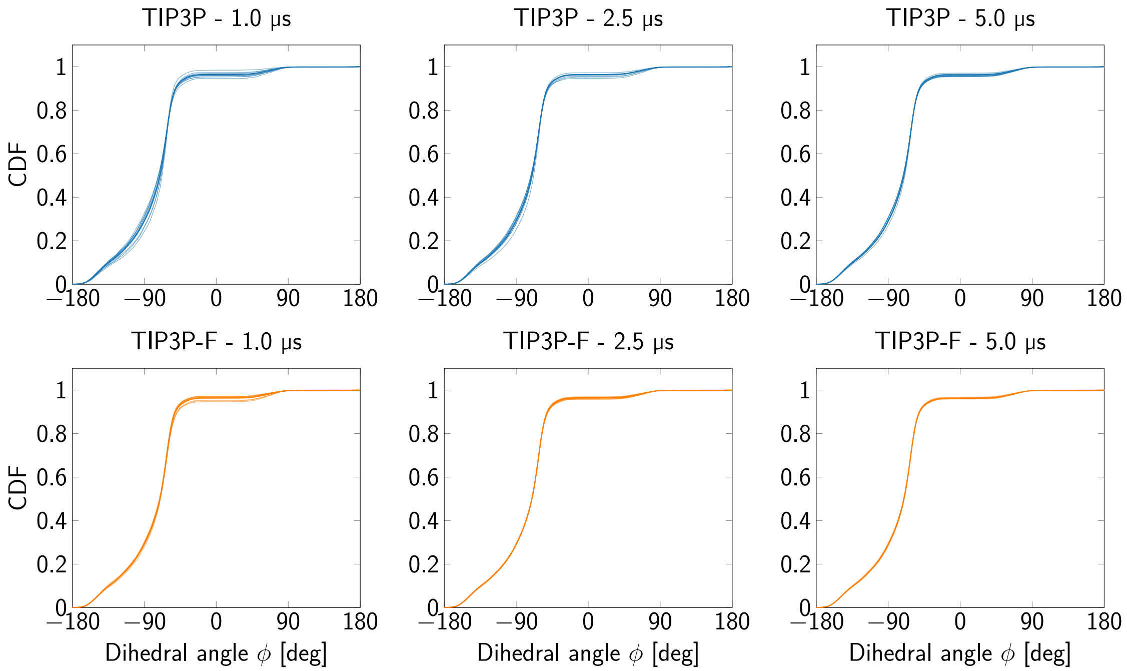

Figure S6: Convergence of the distribution of the central ϕ dihedral angle of the alanine decapeptide. Panels show the cumulative distribution function up to different time points (left to right) in the respective simulations for TIP3P (top) and TIP3P-F water (bottom). Light and dark colored lines show the distributions for different replicates and the final average distribution, respectively.

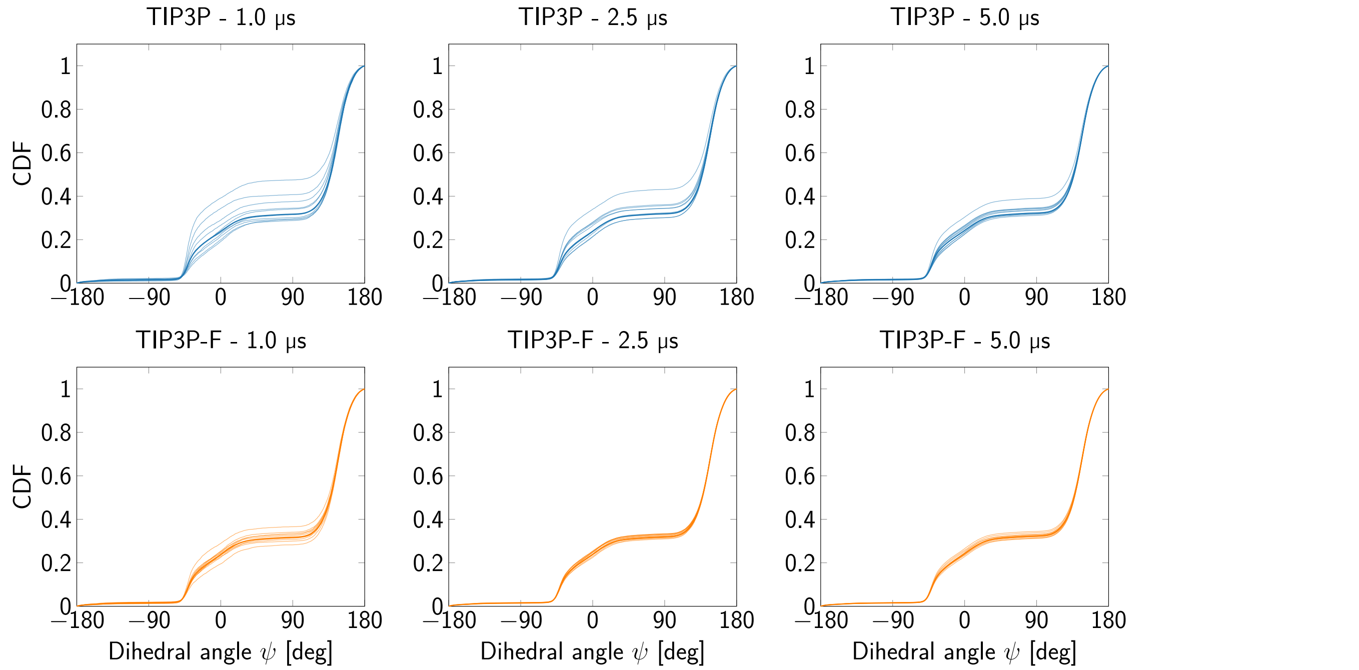

Figure S7: Convergence of the distribution of the central ψ dihedral angle of the alanine decapeptide. Panels show the cumulative distribution function up to different time (left to right) points in the respective simulations for TIP3P (top) and TIP3P-F water (bottom). Light and dark colored lines show the distributions for the different replicates and the final average distribution, respectively.

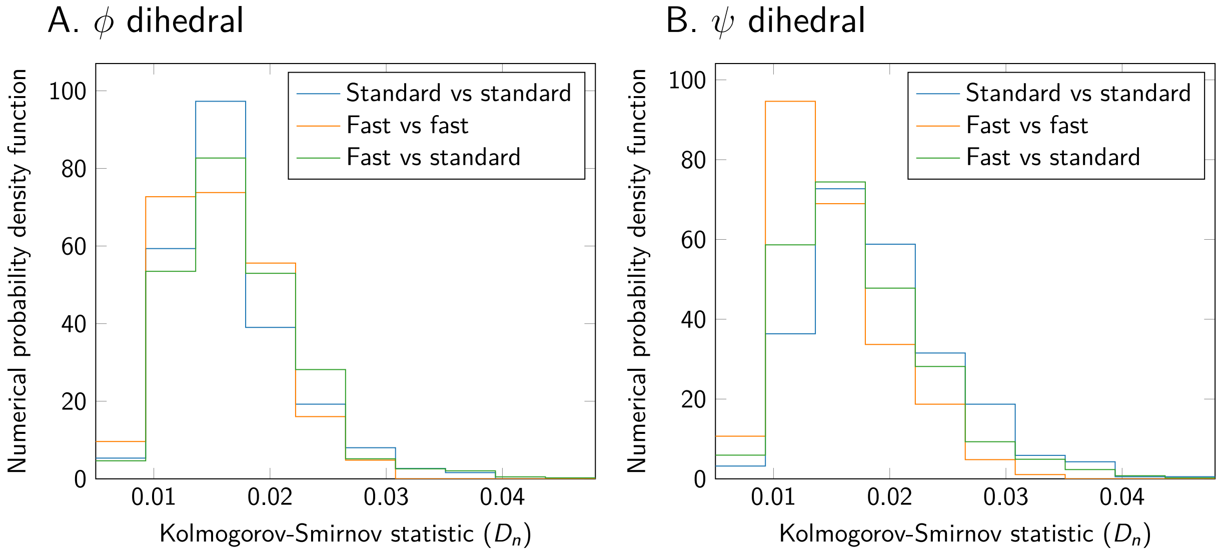

Figure S8: Observed distributions of the Kolmogorov-Smirnov statistic comparing pairs of trajectories of the ϕ and ψ dihedrals in the alanine dipeptide.

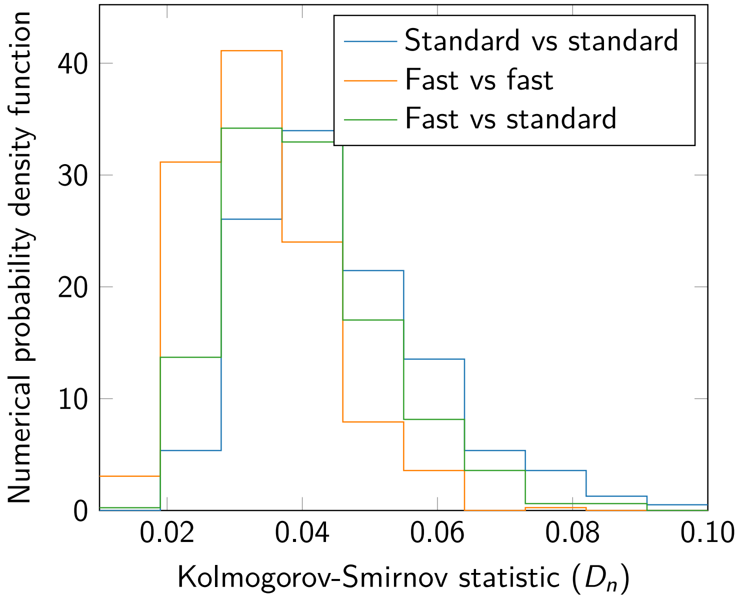

Figure S9: Numerical distribution of the Kolmogorov-Smirnov statistic between the CDFs of the N-terminal to C-terminal distance of the alanine pentapeptide.

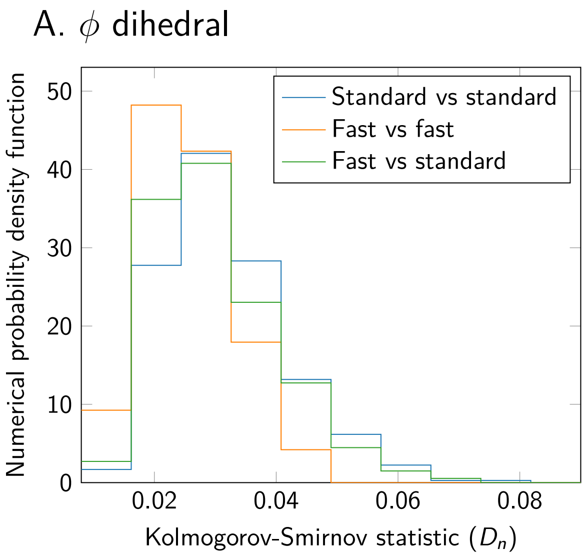

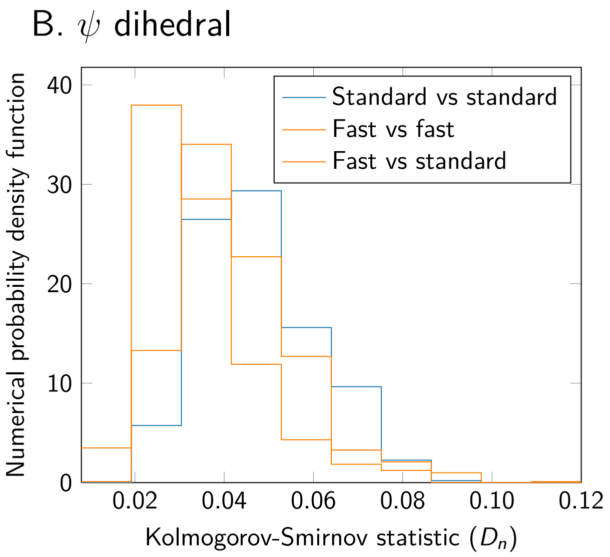

Figure S10: Numerical distributions of the pair-wise Kolmogorov-Smirnov statistic between the CDFs of the Ramachandran angles in the central residue of the alanine pentapeptide.

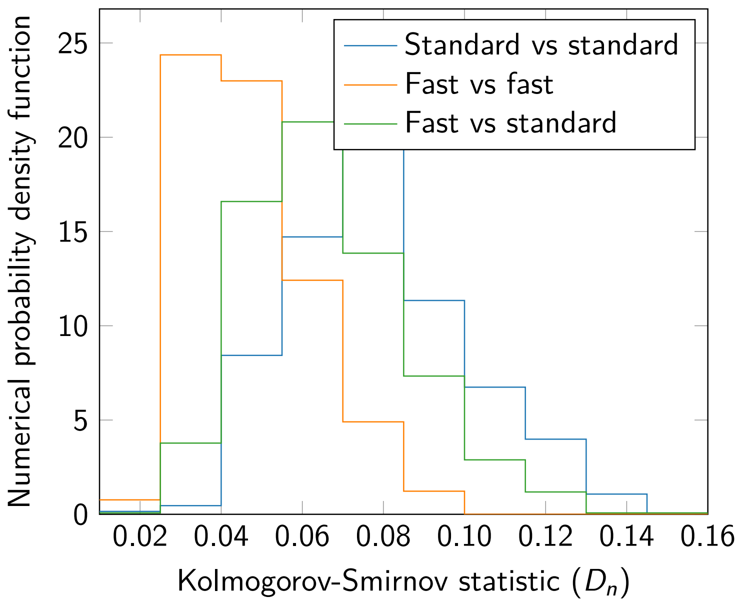

Figure S11: Observed numerical distributions of the pair-wise Kolmogorov-Smirnov statistic between independent molecular dynamics runs, for the N-terminal to C-terminal distance of the alanine decapeptide.

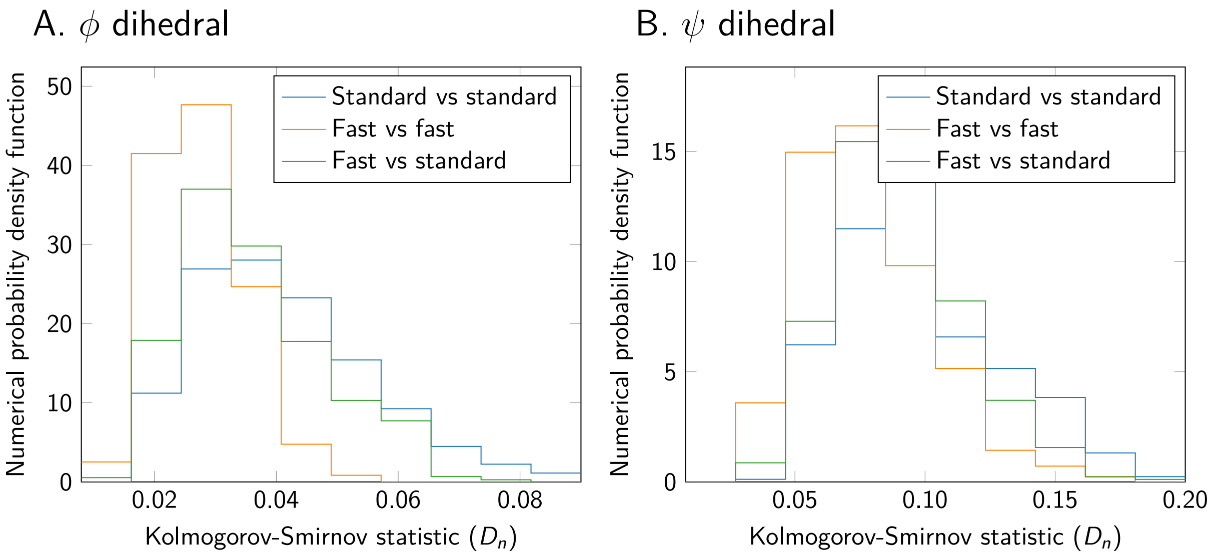

Figure S12: Numerical distributions of the pair-wise Kolmogorov-Smirnov statistic between independent molecular dynamics runs, for the Ramachandran dihedrals of the central residue in the alanine decapeptide.

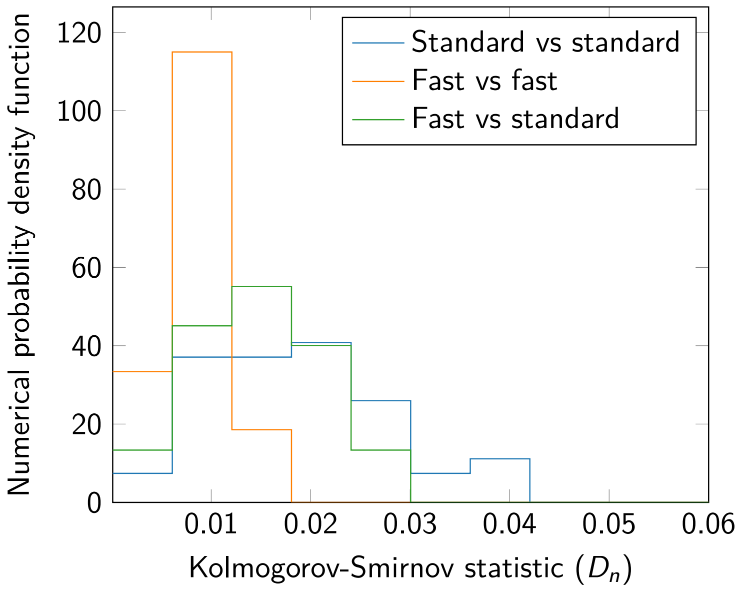

Figure S13: Numerical distributions of the pair-wise Kolmogorov-Smirnov statistic comparing trajectories from independent molecular dynamics runs, for the O5' to O3' distance of the adenine diribonucleotide.

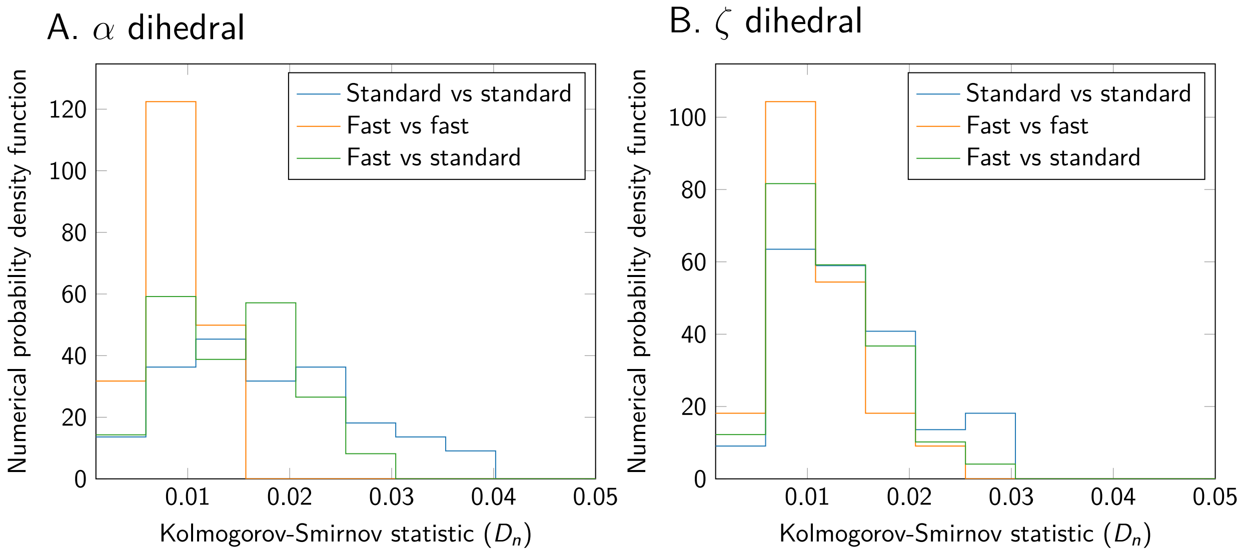

Figure S14: Numerical distributions of the pair-wise Kolmogorov-Smirnov statistic comparing trajectories from independent molecular dynamics runs, for central phosphate dihedrals of the adenine diribonucleotide.

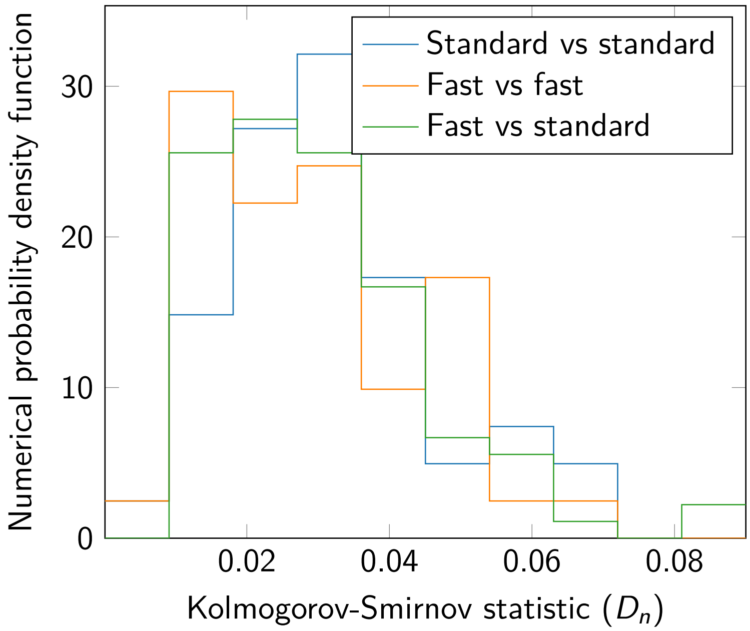

Figure S15: Numerical distributions of the pair-wise Kolmogorov-Smirnov statistic comparing trajectories from independent molecular dynamics runs, for the O5' to O3' distance of the adenine tetraribonucleotide.

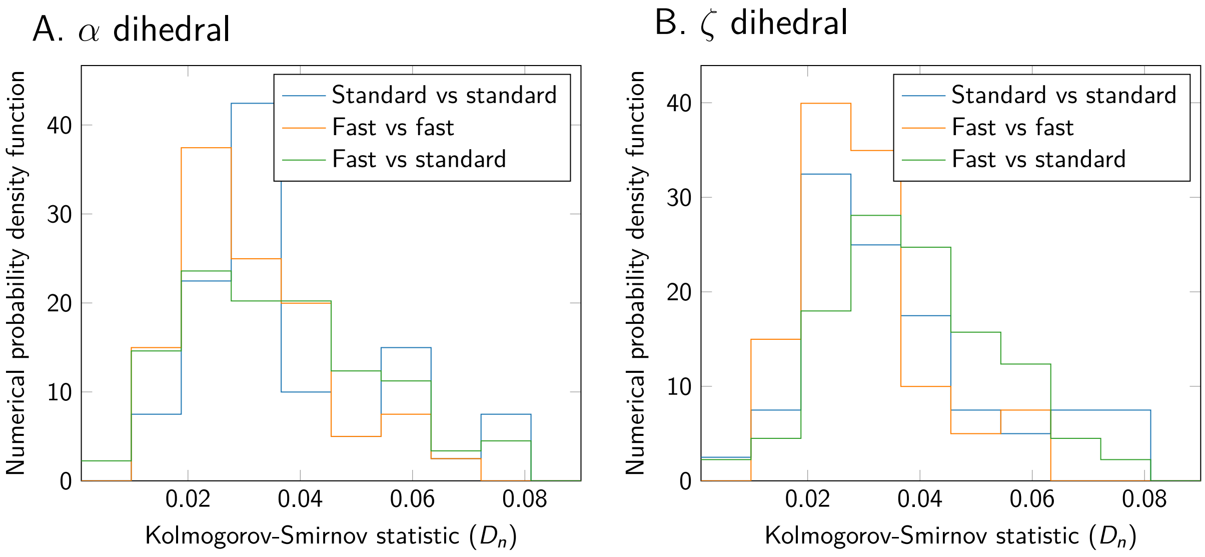

Figure S16: Numerical distributions of the pair-wise Kolmogorov-Smirnov statistic comparing trajectories from independent molecular dynamics runs, for central phosphate dihedrals of the adenine tetraribonucleotide.

References

(S1) Virtanen, P.; Gommers, R.; Oliphant, T. E.; Haberland, M.; Reddy, T.; Cournapeau, D.; Burovski, E.; Peterson, P.; Weckesser, W.; Bright, J.; van der Walt, S. J.; Brett, M.; Wilson, J.; Millman, K. J.; Mayorov, N.; Nelson, A. R. J.; Jones, E.; Kern, R.; Larson, E.; Carey, C. J.; Polat, İ.; Feng, Y.; Moore, E. W.; VanderPlas, J.; Laxalde, D.; Perktold, J.; Cimrman, R.; Henriksen, I.; Quintero, E. A.; Harris, C. R.; Archibald, A. M.; Ribeiro, A. H.; Pedregosa, F.; van Mulbregt, P.; SciPy 1.0 Contributors, SciPy 1.0: Fundamental algorithms for scientific computing in python. *Nat. Meth.* **2020**, *17*, 261–272.

(S2) Sokal, A. In *Functional Integration: Basics and Applications*; DeWitt-Morette, C., Cartier, P., Folacci, A., Eds.; Springer, Boston, MA, 1997; pp 131–192.

(S3) van der Spoel, D.; Berendsen, H. Molecular dynamics simulations of Leu-enkephalin in water and DMSO. *Biophys. J.* **1997**, *72*, 2032–2041.

(S4) Bussi, G.; Tribello, G. A. In *Biomolecular Simulations: Methods and Protocols*; Bonomi, M., Camilloni, C., Eds.; Springer New York: New York, NY, 2019; pp 529–578.
